# *FOXI3* pathogenic variants cause one form of craniofacial microsomia

**DOI:** 10.1038/s41467-023-37703-6

**Published:** 2023-04-11

**Authors:** Ke Mao, Christelle Borel, Muhammad Ansar, Angad Jolly, Periklis Makrythanasis, Christine Froehlich, Justyna Iwaszkiewicz, Bingqing Wang, Xiaopeng Xu, Qiang Li, Xavier Blanc, Hao Zhu, Qi Chen, Fujun Jin, Harinarayana Ankamreddy, Sunita Singh, Hongyuan Zhang, Xiaogang Wang, Peiwei Chen, Emmanuelle Ranza, Sohail Aziz Paracha, Syed Fahim Shah, Valentina Guida, Francesca Piceci-Sparascio, Daniela Melis, Bruno Dallapiccola, Maria Cristina Digilio, Antonio Novelli, Monia Magliozzi, Maria Teresa Fadda, Haley Streff, Keren Machol, Richard A. Lewis, Vincent Zoete, Gabriella Maria Squeo, Paolo Prontera, Giorgia Mancano, Giulia Gori, Milena Mariani, Angelo Selicorni, Stavroula Psoni, Helen Fryssira, Sofia Douzgou, Sandrine Marlin, Saskia Biskup, Alessandro De Luca, Giuseppe Merla, Shouqin Zhao, Timothy C. Cox, Andrew K. Groves, James R. Lupski, Qingguo Zhang, Yong-Biao Zhang, Stylianos E. Antonarakis

**Affiliations:** 1 School of Engineering Medicine, Beihang University, Beijing, 100191 China; 2 Department of Genetic Medicine and Development, University of Geneva Medical Faculty, Geneva, 1211 Switzerland; 3 Jules-Gonin Eye Hospital, Department of Ophthalmology, University of Lausanne, 1004 Lausanne, Switzerland; 4 Department of Molecular and Human Genetics, Baylor College of Medicine, Houston, TX 77030 USA; 5 Laboratory of Medical Genetics, Medical School, University of Athens, Athens, Greece; 6 Biomedical Research Foundation of the Academy of Athens, Athens, Greece; 7 CeGaT GmbH and Praxis für Humangenetik Tuebingen, Tuebingen, 72076 Germany; 8 Molecular Modeling Group, Swiss Institute of Bioinformatics, Lausanne, 1015 Switzerland; 9 Plastic Surgery Hospital, Chinese Academy of Medical Sciences, Beijing, 100144 China; 10 Key Laboratory of Big Data-Based Precision Medicine (Beihang University), Ministry of Industry and Information Technology, Beijing, China; 11 Department of Plastic Surgery, Affiliated Hospital of Xuzhou Medical University, Xuzhou, 221000 China; 12 Medigenome, Swiss Institute of Genomic Medicine, 1207 Geneva, Switzerland; 13 Department of Biotechnology, School of Bioengineering, SRMIST, Kattankulathur, Tamilnadu 603203 India; 14 Department of Neuroscience, Baylor College of Medicine, Houston, TX 77030 USA; 15 Department of Otolaryngology-Head and Neck Surgery, Beijing Tongren Hospital, Capital Medical University, Beijing, China; 16 Anatomy Department, Khyber Medical University Institute of Medical Sciences (KIMS), Kohat, Pakistan; 17 Department of Medicine, KMU Institute of Medical Sciences (KIMS), DHQ Hospital KDA, Kohat, Pakistan; 18 Medical Genetics Division, Fondazione IRCCS Casa Sollievo della Sofferenza, San Giovanni Rotondo, Italy; 19 Department of Medicine, Surgery, and Dentistry, Università University degli of Studi di Salerno, Salerno, Italy; 20 Medical Genetics and Rare Disease Research Division, Pediatric Cardiology, Medical Genetics Laboratory, Neuropsychiatry, Scientific Rectorate, Bambino Gesù Children Hospital, IRCCS, Rome, Italy; 21 Sezione di Genetica Medica, Ospedale ‘Bambino Gesù’, Rome, Italy; 22 Department of Maxillo-Facial Surgery, Policlinico Umberto I, Rome, Italy; 23 Department of Fundamental Oncology, Ludwig Institute for Cancer Research, Lausanne University, Epalinges, 1066 Switzerland; 24 Laboratory of Regulatory & Functional Genomics, Fondazione IRCCS Casa Sollievo della Sofferenza, San Giovanni Rotondo, Italy; 25 Medical Genetics Unit, Hospital Santa Maria della Misericordia, Perugia, Italy; 26 Medical Genetics Unit, University of Perugia Hospital SM della Misericordia, Perugia, Italy; 27 Medical Genetics Unit, Meyer Children’s University Hospital, Florence, Italy; 28 Pediatric Department, ASST Lariana, Santa Anna General Hospital, Como, Italy; 29 Division of Evolution, Infection and Genomics, School of Biological Sciences, University of Manchester, Manchester, UK; 30 Department of Medical Genetics, Haukeland University Hospital, Bergen, Norway; 31 Centre de Référence Surdités Génétiques, Hôpital Necker, Institut Imagine, Paris, France; 32 Department of Molecular Medicine and Medical Biotechnology, University of Naples Federico II, Via S. Pansini 5, 80131 Naples, Italy; 33 Departments of Oral & Craniofacial Sciences and Pediatrics, University of Missouri-Kansas City, Kansas City, MO 64108 USA; 34 Department of Pediatrics, Baylor College of Medicine, Houston, TX 77030 USA; 35 Human Genome Sequencing Center, Baylor College of Medicine, Houston, TX 77030 USA; 36 iGE3 Institute of Genetics and Genomes in Geneva, Geneva, Switzerland

**Keywords:** Genetics, Molecular medicine, Development

## Abstract

Craniofacial microsomia (CFM; also known as Goldenhar syndrome), is a craniofacial developmental disorder of variable expressivity and severity with a recognizable set of abnormalities. These birth defects are associated with structures derived from the first and second pharyngeal arches, can occur unilaterally and include ear dysplasia, microtia, preauricular tags and pits, facial asymmetry and other malformations. The inheritance pattern is controversial, and the molecular etiology of this syndrome is largely unknown. A total of 670 patients belonging to unrelated pedigrees with European and Chinese ancestry with CFM, are investigated. We identify 18 likely pathogenic variants in 21 probands (3.1%) in *FOXI3*. Biochemical experiments on transcriptional activity and subcellular localization of the likely pathogenic *FOXI3* variants, and knock-in mouse studies strongly support the involvement of *FOXI3* in CFM. Our findings indicate autosomal dominant inheritance with reduced penetrance, and/or autosomal recessive inheritance. The phenotypic expression of the *FOXI3* variants is variable. The penetrance of the likely pathogenic variants in the seemingly dominant form is reduced, since a considerable number of such variants in affected individuals were inherited from non-affected parents. Here we provide suggestive evidence that common variation in the *FOXI3* allele in *trans* with the pathogenic variant could modify the phenotypic severity and accounts for the incomplete penetrance.

## Abstract (web summary)

Most cases of craniofacial microsomia are sporadic but familial cases have been reported. Here the authors report that variants in FOXI3 can cause a small fraction of cases with different modes of inheritance including autosomal dominant with reduced penetrance.

## Introduction

In 1952, Maurice Goldenhar in Geneva described a disorder of craniofacial morphogenesis known as Goldenhar syndrome (OMIM 164210)^1,2^. The syndrome is also known as craniofacial microsomia (CFM) and other names such as Oculo-Auriculo-Vertebral spectrum^3^ or hemifacial microsomia. The craniofacial anomalies include ear dysplasia, microtia, and preauricular tags, and originate from the first and second pharyngeal arch-derived tissues (a comprehensive phenotypic description is given here^4^). Most cases are sporadic; however, reports of familial cases suggest either an autosomal dominant (AD) or autosomal recessive (AR) mode of inheritance^4^. The incidence of CFM ranges from 1:3500 to 1:5600 live births^5^. A pharmacological phenocopy of microtia is known to be caused by isotretinoin exposure during pregnancy^6^. In addition, there is a recent hypothesis that cases of malformation syndromes (including CFM) termed “recurrent constellations of embryonic malformations” (RCEM) may also be caused by environmental factors^7^.

A twin study has provided support for a genetic etiology^8^; 60% concordance in monozygotic twins, but only 5% in dizygotic twins. Various chromosomal abnormalities and structural variants have been described in some sporadic cases of CFM^4^. In addition, variants in genes *MYT1*, *VWA1, SF3B2,* and some others have been proposed as causative^9–14^. *SF3B2* is the most common causative gene to date, haploinsufficiency of which accounts for ~3% of sporadic CFM^14^. However, the molecular basis for the majority of CFM remains elusive. Genome-wide association studies on CFM have identified 15 predisposing genomic signals^15,16^, explaining 8% of the phenotypic variation. Recently, one of the GWAS signals, a ∼10-kb interval in the intergenic region between *ROBO1* and *ROBO2* genes has been strongly associated with an increased risk of microtia in Amerindigenous populations. The risk locus contains a polymorphic complex repeat element that is expanded in affected individuals^17^.

Here we show that *FOXI3* pathogenic alleles cause a small but significant fraction of CFM, particularly that with types II and III microtia or ear deformity and preauricular tags. Mouse and cellular models support this contention. The mode of inheritance of *FOXI3*-related CFM can be either AD with reduced penetrance or AR. Further, we provide evidence to support the hypothesis that a combination of rare pathogenic *FOXI3* alleles in *trans* with a common *FOXI3* haplotype could explain both the penetrance and the variable expressivity of CFM in a compound inheritance gene dosage model.

## Results

### *FOXI3* likely pathogenic variants in a Pakistani CFM family

**Fig. 1:**
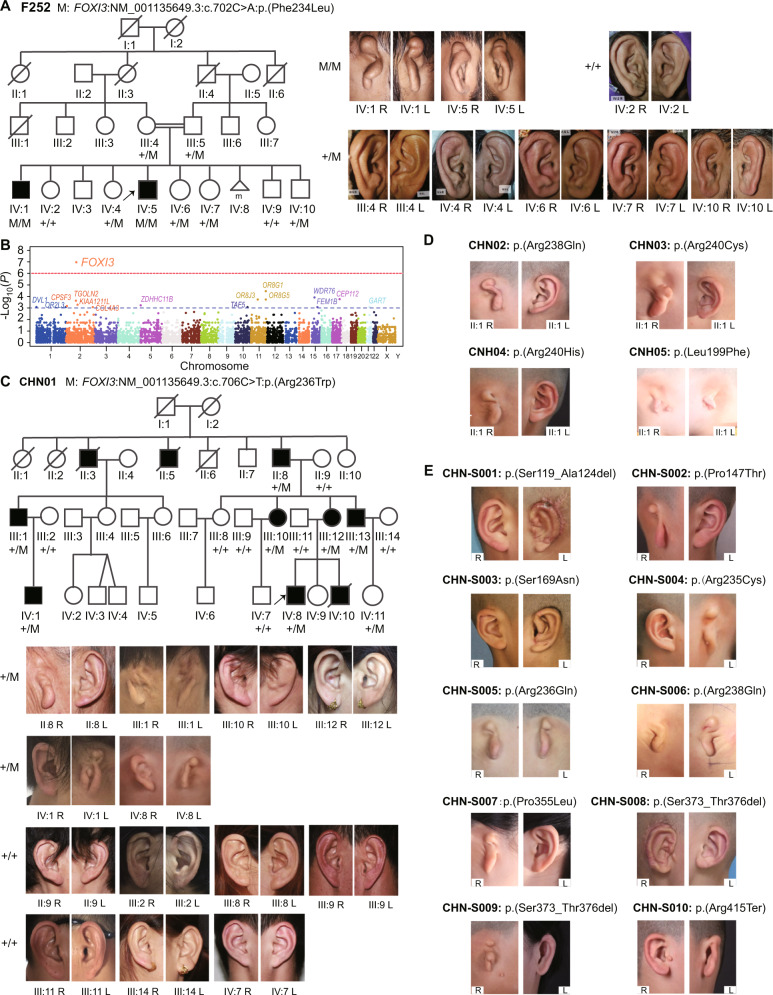
Identification of *FOXI3* as the pathogenic gene for CFM. **A** The Pakistani consanguineous pedigree F252 with the likely pathogenic FOXI3: p.(Phe234Leu) variant. Photographs of the microtia type III of both ears of affected individuals IV:1 and IV:5. The bottom panel depicts the ears of additional members of the F252 family. The Sanger sequences are shown in Supplementary Fig. 1. M depicts the mutant allele, and + the normal allele. **B** Family-based gene-level association on rare LoF (Loss-of-Function) variants from 48 CFM Chinese families. The blue dashed line indicates the significance threshold of 1E-03, and the red dashed line indicates the Bonferroni correction threshold of 1.03E-06. Source data are provided as a Source Data file. **C** The pedigree, genotype, and external ear phenotype for the Chinese CFM family CHN01. M depicts the mutant allele, and + indicates the normal allele. **D** The genotype and external auricular phenotype for the four Chinese CFM families. **E** The genotype and external auricular phenotype of the sporadic Chinese patients.

As part of an autozygosity project^18^, a Pakistani family F252 was ascertained with two affected siblings with type III microtia and deafness; the parents were first cousins (Fig. 1A and Supplementary Table 1). Whole exome sequencing (WES) identified a homozygous *FOXI3* variant (NM_001135649.3):c.702C>A p.(Phe234Leu) as likely causative (Supplementary Fig. 1A). The unaffected parents, and 4 of 6 non-affected siblings, were heterozygous carriers. This family provided evidence for an AR inheritance for this form of CFM.

### *FOXI3* likely pathogenic variants in Chinese CFM families

The laboratory in Beihang University, independently recruited 159 individuals in 48 Chinese families with CFM and performed WES on all of them; clinical phenotypes are summarized in Supplementary Table 1 and Supplementary Table 2. Family-based gene burden tests on the rare non-synonymous and Loss-of-Function (LoF) variants found the *FOXI3* gene was the only genome-wide significant one (*P* = 1.08E-07) (Fig. 1B and Supplementary Table 3). The largest Chinese CFM family CHN01 shows an AD inheritance pattern with a deleterious heterozygous variant of *FOXI3*:c.706C>T p.(Arg236Trp) co-segregating with CFM (Fig. 1C and Supplementary Fig. 1B). The phenotypes vary greatly, and range from an absence of the posterior crus of the antihelix to bilateral type III microtia and micrognathia in CHN01 (Fig. 1C and Supplementary Table 1).

**Fig. 2:**
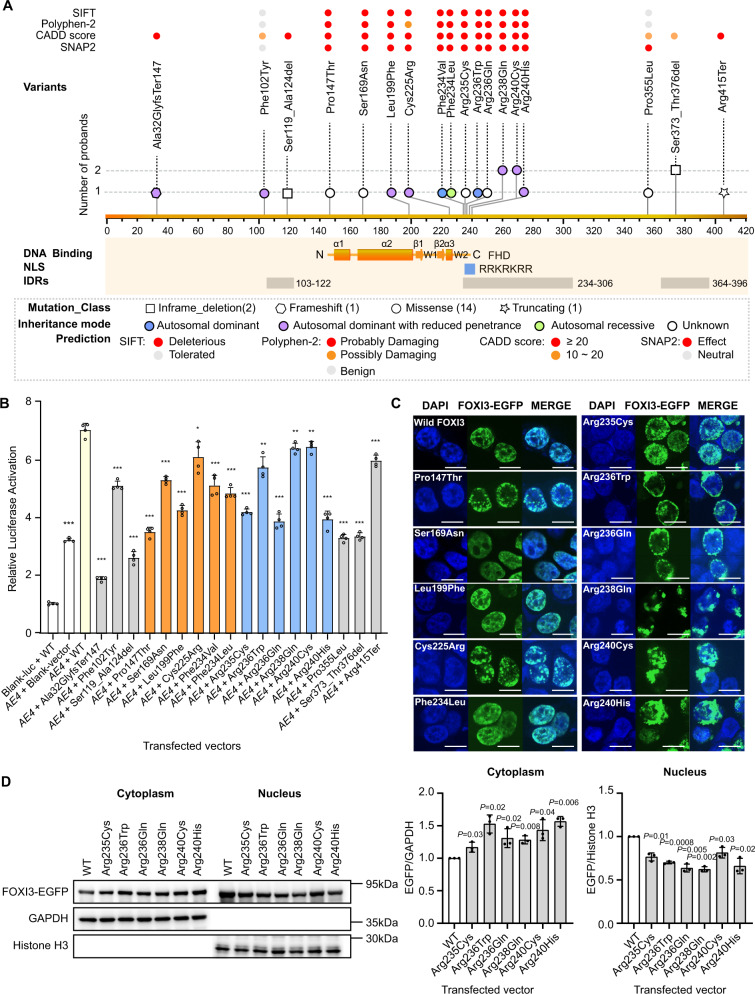
The effect of FOXI3 variants with in silico prediction and in vitro experiments. **A** The 18 identified variants on FOXI3. The predictions of the variants are classified by bins and shown on the top of each variant. The variants are classified by the type of inframe_deletion (square), frameshift (hexagon), missense (circle), and truncating (star), and the inheritance mode of autosomal dominant (blue) or with reduced penetrance (purple), autosomal recessive (green), and unknown (white). The variants of p.(Arg238Gln), p.(Arg240Cys), and p.(Ser373_Thr376del) are detected in two probands and the rest are in one proband. **B** Effects of the 18 variants on the transactivation of FOXI3 in HEK293T cells (*n* = 4/group). Gray Bars: intrinsically disordered regions (IDRs), orange bars: forkhead domain (FHD), blue bar: nuclear localization signal (NLS). *P < 0.05, **P < 0.01, ***P < 0.001. Each experiment was repeated at least three times. **C** The localization and distribution of wild type or mutant FOXI3-EGFP fusion proteins in HEK-293T cells. DAPI counterstain (blue) shows the location of the nucleus. EGFP (green fluorescent protein) shows the subcellular localization of the FOXI3-EGFP fusion protein. Wild-type FOXI3 is almost completely transferred into the nucleus and uniformly distributed. A majority of FOXI3 with mutant NLS is blocked outside the nucleus; FOXI3 with mutant FHD tends to form intranuclear aggregation; most of FOXI3 with mutant IDR is similar to Wild-type FOXI3. Scale bar = 10 μm. Each experiment was performed in triplicate and repeated at least three times. **D** Representative western blots of FOXI3-EGFP with mutant NLS. Immunoprecipitation on the EGFP-tagged FOXI3 using antibody of EGFP and detect the amount of FOXI3-EGFP in the nucleus and cytoplasmic fractions of HEK293T cells (*n* = 3 biologically independent experiments). Compared to the wild type, the mutant FOXI3-EGFP fusion protein is significantly trapped in the cytoplasm and the amount of mutant FOXI3-EGFP is significantly reduced in the nucleus. Significant differences between two groups (wild type and each mutant) were determined by unpaired Student’s *t*-test (two-tailed). Data are presented as the means ± s.d. Source data are provided as a Source Data file.

There were additional *FOXI3* likely pathogenic variants in 4 of the 48 Chinese CFM families: FOXI3:p.(Leu199Phe), p.(Arg238Gln), p.(Arg240Cys), and p.(Arg240His) (Fig. 1D, Supplementary Fig. 2A, and Table 1). A further 9 *FOXI3* variants were found by targeted-capture sequencing in a cohort of 498 additional sporadic CFM patients: 3 variants p.(Arg235Cys), p.(Arg236Gln), and p.(Arg238Gln) are located in the nuclear localization signal (NLS); 2 variants p.(Pro147Thr) and p.(Ser169Asn), are located in the Forkhead domain (FHD); 2 inframe deletions of p.(Ser119_Ala124del) and p.(Ser373_Thr376del), and 2 variants of p.(Pro355Leu) and p.(Arg415Ter) map in the intrinsically disordered regions (IDRs) of FOXI3 (Fig. 1E, Fig. 2A, and Table 1). Supplementary Table 4 lists the variants in known candidate genes related to syndromes with microtia found in this cohort.

**Table 1:** Likely pathogenic rare variants within the *FOXI3* gene identified from patients (CFM or Goldenhar syndrome)

Family ID	Proband ID	Chromosome Position (hg38)	Reference	Alternative	Consequence	*FOXI3* cHGVS NM_001135649.3	FOXI3 pHGVS	In silico predictions	gnomAD_Allele_Frequency (v3.1.2)
EUR04	EUR04 (SEA15423)	chr2:88452445	G	GG	frameshift	c.92dupG	p.(Ala32GlyfsTer147)	CADD: 23.0	absent
EUR05	EUR05 (SEA15566)	chr2:88452231	A	T	missense	c.305T>A	p.(Phe102Tyr)	CADD: 14.07 SIFT: 0.96 Polyphen2: 0	absent
CHN-S001	CHN-S001	chr2:88452164	CGCGGGCGCGGCGGGCGAG	C	inframe deletion	c.354_371del	p.(Ser119_Ala124del)	CADD: 19.88	0.0000199
CHN-S002	CHN-S002	chr2:88452097	G	T	missense	c.439C>A	p.(Pro147Thr)	CADD: 28.3 SIFT: 0 Polyphen2: 1	absent
CHN-S003	CHN-S003	chr2:88452030	C	T	missense	c.506G>A	p.(Ser169Asn)	CADD: 27.4 SIFT: 0 Polyphen2: 0.994	absent
CHN05	CHN05	chr2:88451941	G	A	missense	c.595C>T	p.(Leu199Phe)	CADD: 29.8 SIFT: 0 Polyphen2: 1	absent
EUR03	EUR03 (SEA15422)	chr2:88448797	A	G	missense	c.673T>C	p.(Cys225Arg)	CADD: 26.4 SIFT: 0.02 Polyphen2:0.599	absent
EUR02	EUR02(SEA15533)	chr2:88448770	A	C	missense	c.700T>G	p.(Phe234Val)	CADD: 29.1 SIFT: 0 Polyphen2: 0.992	absent
F252	IV: 5	chr2:88448768	G	T	missense	c.702C>A	p.(Phe234Leu)	CADD: 26.4 SIFT: 0 Polyphen2: 0.896	0.00001314
CHN-S004	CHN-S004	chr2:88448767	G	A	missense	c.703C>T	p.(Arg235Cys)	CADD: 32 SIFT: 0 Polyphen2: 0.998	absent
CHN01	IV: 8	chr2:88448764	G	A	missense	c.706C>T	p.(Arg236Trp)	CADD: 23.9 SIFT: 0.03 Polyphen2: 0.899	absent
CHN-S005	CHN-S005	chr2:88448763	C	T	missense	c.707G>A	p.(Arg236Gln)	CADD: 28.7 SIFT: 0 Polyphen2: 0.899	0.00000657
CHN02	II: 2	chr2:88448757	C	T	missense	c.713G>A	p.(Arg238Gln)	CADD: 28.8 SIFT: 0.04 Polyphen2: 1	absent
CHN-S006	CHN-S006	chr2:88448757	C	T	missense	c.713G>A	p.(Arg238Gln)	CADD: 28.8 SIFT: 0.04 Polyphen2: 1	absent
CHN03	II: 2	chr2:88448752	G	A	missense	c.718C>T	p.(Arg240Cys)	CADD: 29.1 SIFT: 0 Polyphen2: 0.993	absent
EUR01	EUR01 (SEA9213)	chr2:88448752	G	A	missense	c.718C>T	p.(Arg240Cys)	CADD: 29.1 SIFT: 0 Polyphen2: 0.993	absent
CHN04	II: 2	chr2:88448751	C	T	missense	c.719G>A	p.(Arg240His)	CADD: 28.2 SIFT: 0 Polyphen2: 0.993	0.00002628
CHN-S007	CHN-S007	chr2:88448406	G	A	missense	c.1064C>T	p.(Pro355Leu)	CADD: 12.73 SIFT: 0.09 Polyphen2: 0.003	0.00002094
CHN-S008	CHN-S008	chr2:88448341	CGGTGCTATTGCT	C	inframe deletion	c.1117_1128del	p.(Ser373_Thr376del)	CADD: 14.11	0.00003245
CHN-S009	CHN-S009	chr2:88448341	CGGTGCTATTGCT	C	inframe deletion	c.1117_1128del	p.(Ser373_Thr376del)	CADD: 14.11	0.00003245
CHN-S010	CHN-S010	chr2:88448227	G	A	stop gained	c.1243C>T	p.(Arg415Ter)	CADD:38	0.000006574
EUR06	EUR06 (SEA15421)	chr2:87317229-89306982	+	-	SV deletion	chr2: 87317229-89306982 deletion	/	/	/

### *FOXI3* likely pathogenic variants in European CFM families

The Geneva laboratory screened the *FOXI3* gene by Sanger sequencing or WES in 124 CFM probands of European ancestry; 6 probands with heterozygous rare non-synonymous or LoF, likely pathogenic variants were identified (Table 1, Supplementary Table 1, and Supplementary Fig. 2B). The phenotype of 5 European CFM families was CFM with isolated microtia type II or type III (family EUR01 is shown in Supplementary Fig. 2C). The variants identified were p.(Ala32GlyfsTer147), p.(Phe102Tyr), p.(Cys225Arg), p.(Phe234Val), and p.(Arg240Cys) were heterozygous in the five probands, respectively (Table 1). Notably, these variants were inherited in each case from an asymptomatic individual (EUR01, EUR03, EUR04, and EUR05) or transmitted from a mildly affected parent (EUR02) (Supplementary Fig. 2B), indicating autosomal dominant inheritance with reduced penetrance. In family EUR01, two asymptomatic brothers of the proband also inherited the mutant p.(Arg240Cys) allele.

In order to test the hypothesis that a modifier *FOXI3*-related variation in *trans* with a pathogenic FOXI3 allele contributes to the phenotype in the seemingly autosomal dominant pedigrees presented above, we studied samples from a proband of family EUR06 previously described with type III microtia, and a 2.06 Mb deletion that included *FOXI3* inherited from the asymptomatic father^19^. We used primary cultured fibroblasts (FFF0062013 cell line) from this case to identify a potentially contributing variant in the remaining *FOXI3* allele. WGS with short reads, did not identify any candidate non-coding variants within the *FOXI3*-containing TAD. WGS long-read Nanopore did not reveal any structural variant at the remaining *trans FOXI3* locus. Whole-genome optical mapping also validated the large 2.06 Mb deletion (Supplementary Fig. 3) and did not reveal any abnormality in the remaining copy of the *FOXI3* locus. We were able to determine the haplotype of the polymorphic sites of the remaining *FOXI3* allele. This haplotype contains two linkage disequilibrium (LD) blocks, and is common in Europeans (12.9%) and East Asians (19.7%) (data from LDlink 5.1 https://ldlink.nci.nih.gov/) (Supplementary Fig. 4). No quantitative trait loci (QTLs) were identified in GTEx due to undetectable expression of *FOXI3* in adult tissues^20–22^. This haplotype is discussed further below.

### The contribution of FOXI3 mutations to CFM

The frequency of the *FOXI3* causative variants in CFM patients of European ancestry is 5 out of 124 (4.0%); the case of EUR06 was not counted since it has been described in the literature and the total number of CFM patients studied was not mentioned. In the 48 unrelated CFM families of Chinese ancestry, 5 had *FOXI3* pathogenic variants (10.4%). In the 498 additional Chinese CFM cases, there were ten patients with pathogenic *FOXI3* variants (2.0%). Thus, *FOXI3* pathogenic variants account for 3.1% of CFM cases of European and Chinese ancestry combined (21 of 670 cases). The frequency of all *FOXI3* variants with a CADD score of >20 in the BRAVO database^23^ (https://bravo.sph.umich.edu/freeze8/hg38) is 0.34% (456 heterozygous in 132,345 samples) which is 10 times less frequent than in CFM (*χ*^*2*^ test *P* value 10^−5^).

### In silico analysis of FOXI3 deleterious variants

Figure 2A depicts the mapping of the 18 identified likely pathogenic variants including the 14 non-synonymous variants in *FOXI3*; 6 map within the FHD, further 6 within the NLS, and 2 within the IDR domains. These variants were predicted to be damaging to FOXI3 according to the scores of SIFT (Sorting Intolerant From Tolerant), Polyphen (Polymorphism Phenotyping v2), CADD (Combined Annotation Dependent Depletion), or SNAP2 (Fig. 2A).

We performed 3D modeling based on the structures of the related human FOXC2 (PDB ID: 6AKO)^24^ or Foxk1A (PDB ID: 2C6Y)^25^ (Supplementary Fig. 5A). The modeled FOXI3:DNA complex covers the FHD and NLS which harbor 12 variants distributed at 9 sites (Supplementary Fig. 5B, C). The p.(Pro147Thr), p.(Cys225Arg), and p.(Phe234Val) would decrease free energy by more than 1.6 kcal/mol and, using Foldx software^26^, are predicted to affect the stability of FOXI3 (Supplementary Fig. 5D). In the 3D modeled FOXI3, the Cys225 was predicted to form a disulfide bond with Cys202, while Arg225 could not. Phe234 was on the C-terminal end of the Forkhead domain and was predicted to interact with Ala152 and Ala155 of alpha-helix 2 and with Phe229, forming a hydrophobic cluster. The mutant Val234 would change the hydrophobic cluster and destabilize the structure of FOXI3 (Supplementary Fig. 5E). The p.(Ser169Asn) and p.(Leu199Phe) are both in the alpha-helix and cannot form hydrogen bonds with Thr167 and His195, respectively, which may affect the stability of FOXI3 (Supplementary Fig. 5E). The positively charged residues of Arg235, Arg236, Arg238, and Arg240 are all within the conserved NLS domain (Supplementary Fig. 6A) and were predicted to affect the ability of FOXI3 to enter the nucleus (Supplementary Fig. 6B). Each of these variants also conforms to the CG to TG hypermutation rule^27^. As with variants in other NLS sequences, these variants were also predicted to perturb binding DNA as the substitutions would break the hydrogen bonds with phosphate moieties of the DNA backbone; this is consistent with the free energy calculation prediction (Δ*G*mut − Δ*G*wt < 1.6 kcal/mol and ΔΔ*G*_(monomer)_ − ΔΔG_(complex)_ > 0.25; Supplementary Fig. 5D).

### In vitro analysis of FOXI3 deleterious variants

We investigated the effects of all 18 likely pathogenic variants on the transcriptional activity of FOXI3. We co-transfected wild-type or mutant *FOXI3* with the *AE4* promoter luciferase reporter^28,29^ in HEK-293T cells and found that the luciferase activity was reduced for all mutations compared to wild-type *FOXI3* (Fig. 2B).

We transfected EGFP-tagged *FOXI3* into HEK-293T cells followed by immunostaining to visualize the subcellular localization of mutant FOXI3. The wild-type FOXI3 was predominantly localized in the nucleus; the mutant FOXI3 were (i) barely or partially entering the nucleus, and aggregating in the cytoplasm and nucleus (p.(Arg235Cys), p.(Arg236Trp), p.(Arg236Gln), p.(Arg238Gln), p.(Arg240Cys), p.(Arg240His); (ii) mostly entering the nucleus but aggregating into granular foci (p.(Pro147Thr), p.(Cys225Arg), p.(Phe234Val); (iii) no difference from the wild-type (all other FOXI3 variants) (Fig. 2C and Supplementary Fig. 7). Western blot analysis on EGFP-tagged FOXI3 from nuclear and cytoplasmic fractions again demonstrated that the NLS variants significantly reduce the amount of FOXI3 in the nucleus (Fig. 2D).

### Mouse models with *FOXI3* variants

**Fig. 3:**
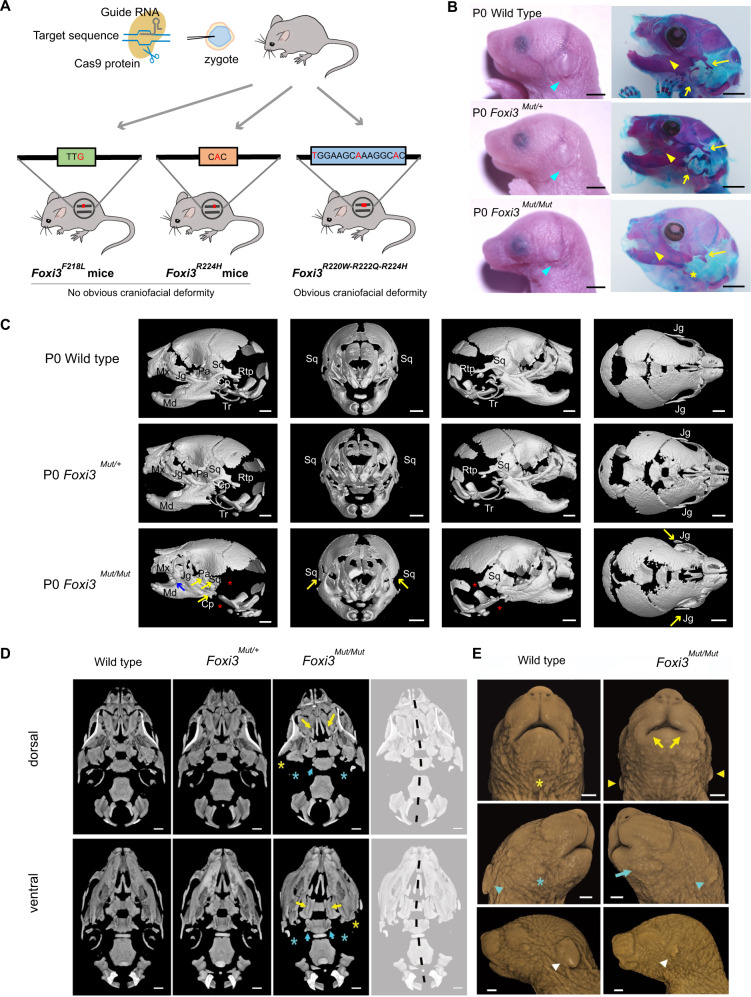
Generation and characterization of FOXI3 mutant mice. **A** A schematic illustration of the generation of Foxi3 mutant mice. Three mouse lines are created including *Foxi3*^*F218L*^, *Foxi3*^*R224H*^, and *Foxi3*^*R220W-R222Q-R224H*^. **B** Craniofacial differences of wild-type, heterozygous, and homozygous *Foxi3*^*R220W-R222Q-R224H*^ neonatal mice (*n* = 3 for each genotype). Comparing with wild type or heterozygotes, the cyan arrows indicate homozygotes with small pinnae, and various overt cranioskeletal defects, including abnormal middle and inner ear bones, syngnathia (mandible and maxilla fusion), and anomalous squamosal development (yellow arrows). The left panel was photographed with bright field; the right panel of mice were stained with Alizarin Red and Alcian Blue. Scale bar = 2 mm. **C** Asymmetric presentation of cranioskeletal features in the neonatal *Foxi3*^*R220W-R222Q-R224H*^ homozygous mice (*n* = 4 for each genotype). Micro-CT images show variable development of the jugal bone, and almost complete loss of the squamous bone in one or both sides in homozygous neonates. The blue arrow indicates the fusion of the mandible (Md) to the maxilla (Mx) in a homozygote. The yellow arrow indicates the poorly developed squamosal (Sq), jugal (Jg), palatine (Pt), and condylar process (Cp). The red asterisk (*) indicates the retrotympanic process (Rtp) and the tympanic ring (Tr) that are absent or severely hypoplastic in homozygotes. **D** Dorsal and ventral views of the cranial base show the absent or hypoplastic bony ear structures (noted in (**C**); asterisked) as well as hypoplasia of the lateral pterygoids of the basisphenoid (cyan arrows) and cleft palate (yellow arrows, dorsal view), with ventrally protruding palatine bones (yellow arrows, ventral view) of newborn triple-mutant mice. Dashed black line denotes the asymmetry of the skull centered on the cranial base. **E** Surface rendering of the facial soft tissue from the same micro-CT scans highlights the severe left-side microtia in the homozygote (white and cyan arrowheads) and asymmetry in positions of the pinnae (yellow arrowheads). Homozygotes exhibit microstomia that is asymmetric in presentation (yellow arrows) and, variably, a row of whisker follicles on the ‘mandible’ (blue arrow) for the newborn triple-mutant mice. Scale bar = 1 mm in (**C**), (**D**), and (**E**). P0: Postnatal day 0.

We produced mouse lines with Foxi3 mutations considering the following criteria: 1) mutations should be located in conserved regions with the highest mouse-human homology, thus those within the FHD domain and NLS (97.9% between human and mouse) were preferred (Supplementary Fig. 8A). 2) mutations in regions such as the NLS of FOXI3 were preferred (*P* = 9.358E-8, Chi-square test). 3) mutations observed in the largest CFM pedigrees (Fig. 1A, C, E, and Supplementary Fig. 8B). 4) mutations that severely affected the transcriptional activity or that severely disturb FOXI3 entering the nucleus. Finally, we produced two mouse lines within the C-terminal end of FHD and the NLS: *Foxi3*^*R224H*^ equivalent to human p.(Arg240His) and *Foxi3*^*F218L*^ equivalent to human p.(Phe234Leu) (Fig. 3A, Supplementary Fig. 9A and 10A).

*Foxi3*^*F218L/F218L*^ and *Foxi3*^*R224H/R224H*^ mice did not show any obvious pathological phenotypes (Supplementary Fig. 10D and Supplementary Fig. 9C). We crossed *Foxi3*^*F218L/F218L*^ to previously produced *Foxi3*^*+/-*^ mice (without abnormalities)^30,31^ to create *Foxi3*^*F218L/-*^ animals to investigate the effect of the mutation. These mice failed to thrive after birth; were underweight, exhibited respiratory abnormalities, and died between birth and day 5. *Foxi3*^*F218L/-*^ mice did not show defects in the external, middle, or inner ears, the mandible, or the maxilla. However, *Foxi3*^*F218L/-*^ mice had absent thymus previously observed in *Foxi3*^*–/–*^ mice^32^ (Supplementary Fig. 10D).

Although we had shown that the human FOXI3 p.(Arg240His) variant is within the NLS and disturbs FOXI3 entering the nucleus (Fig. 2 C, D), we tested whether its homologous variant in Foxi3 p.(Arg224His), as well as all the other five amino acid sites in the NLS, also had the same effect. Surprisingly, transfected EGFP-tagged mutant Foxi3 predominantly entered the nucleus (Supplementary Fig. 11), which differs from the human homologous mutant FOXI3 (Fig. 2C). This phenomenon can be explained by either an artifact of the HEK-293T transfection assay, or a real difference between the nuclear localization requirements of the mouse and human mutant proteins. The latter was considered the likely reason since the Foxi3^*R224H/R224H*^ mice did not show any obvious pathological phenotypes (Supplementary Fig. 9C).

### Mouse model with NLS dysfunction

We found that 6 of 18 identified mutations were in the NLS, which represents a significant enrichment (*P* = 9.358E-8, Chi-square test) since the NLS is comprised of only 7 amino acids (the full length of FOXI3 is 420 AA). Thus, we tried to produce a mouse model with NLS dysfunction to recapitulate the CFM phenotype. We introduced additional identified *FOXI3* variants in the NLS and found that two-point mutant Foxi3 still has the ability to enter the nucleus but that triple-point mutant Foxi3 was unable to enter the nucleus (Supplementary Fig. 11). We, therefore, produced a triple-mutant knock-in mouse line carrying p.(Arg220Trp), p.(Arg222Gln), and p.(Arg224His) (equivalent to human FOXI3 p.(Arg236Trp), p.(Arg238Gln), and p.(Arg240His)), named the triple-mutant model (Fig. 3A and Supplementary Fig. 9B, D).

The birth weight and length of the homozygous neonatal (P0) triple-mutant mice (*n* = 9) were significantly smaller than wild-type (*n* = 7) or heterozygous (*n* = 15) mice (Supplementary Fig. 9E). Homozygous triple-mutant mice die immediately after birth. They show an underdeveloped mandible and a complete absence of the external ear (pinna), replaced by small pre-auricular tags (Fig. 3B and Supplementary Fig. 9F). The heterozygous P0 mice only show a hyperplastic bone between the unfused frontal bones which was also observed in P0 homozygous mice (Fig. 3C). Neonatal homozygous mice revealed a partially truncated mandible and fusion of the upper and lower jaws (syngnathia) and anomalies of various bones of the ear (Fig. 3B); the tympanic ring, malleus, and incus were missing and the stapes were partially developed (Supplementary Fig. 9F).

Micro-CT scans show asymmetric craniofacial defects in homozygous *Foxi3*^*R220W-R222Q-R224H*^ mice. Bony anomalies include poorly developed squamosal (Sq) and jugal (Jg) bones, absent or severely hypoplastic retrotympanic process and tympanic ring (Fig. 3C), hypoplasia of the lateral pterygoids of the basisphenoid and cleft palate in 50% of homozygotes (*n* = 4) (Fig. 3D). All homozygous neonates showed asymmetry of the skull. The pinna varied from severe (grade 4) to grade 2 microtia (Supplementary Fig. 12). Auricular size in homozygotes ranged between 35 and 70% of the wildtype. All homozygotes also exhibited asymmetric microstomia (Fig. 3E). A row of whisker follicles on the “mandible” was evident in one in four homozygotes strongly suggesting the syngnathia is a result of a partial mandibular to maxilla transformation, similar to that in Auriculocondylar syndrome^33^.

### A *FOXI3* region *trans* haplotype as a determinant of reduced penetrance

**Fig. 4:**
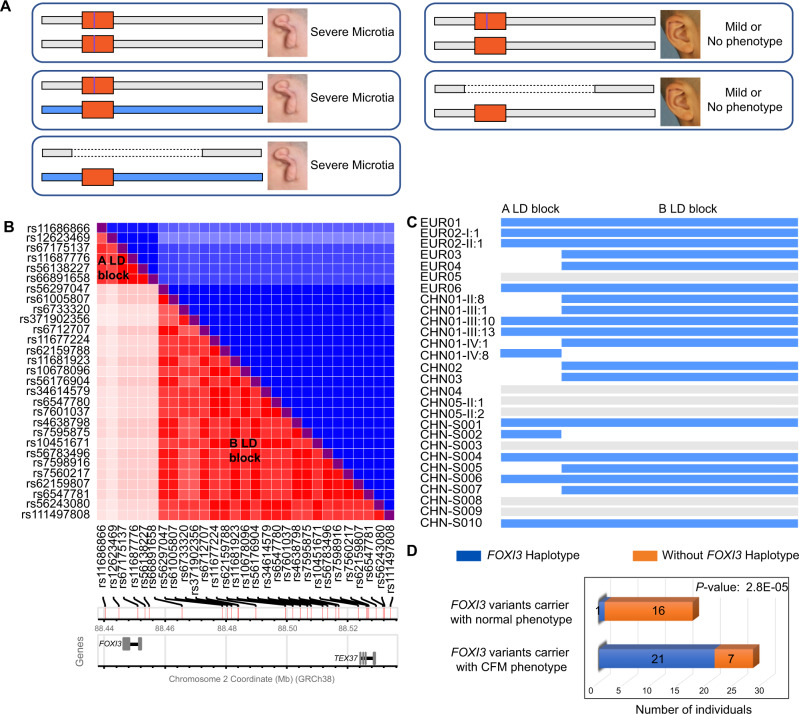
Presumed FOXI3 haplotype that modifies the clinical severity of pathogenic FOXI3 variants. **A** The hypothesis of FOXI3 haplotype for the reduced penetrance of CFM with microtia types II or III. The graph shows three possible genotype combination for CFM with microtia. The orange rectangular box indicates FOXI3 genes in the human genome, the purple line means a pathogenic variant allele. The blue rectangular depicts the presumed modifier *trans* FOXI3 haplotype while the gray rectangular is the common haplotype. The dashed line indicates the missing haplotype. **B** Linkage disequilibrium (LD) blocks A and B (see text) of polymorphic sites shown in the *y*- and *x*-axis (data from Europeans and Chinese). The *x*-axis also depicts a schematic representation of the map position of the polymorphic sites along chromosome 2 (GRCh38). The blue part is the D’ and the red is the *r*^2^ statistics. **C** Presence of the presumed modifier haplotype of A LD block and/or B or both in the DNA of individuals with microtia type III or preauricular tags described in this study. **D** Chi-square test for the *trans* FOXI3 haplotype in CFM and normal individuals. The blue column square indicates the individual who carried FOXI3 variants, as well as trans FOXI3 haplotype, and the orange column square, indicates the individual who carried FOXI3 variants but without *trans* FOXI3 haplotype.

The study of the pedigrees described above demonstrated a reduced *penetrance* for CFM with microtia types II or III. One molecular mechanism of the penetrance could be the nature of the *FOXI3* allele in trans with the pathogenic allele. We propose the following hypothesis (Fig. 4A): (i) homozygotes for a pathogenic variant allele show microtia (pedigree F252). We term this genotype combination *FOXI3 Mut/FOXI3 Mut*; (ii) heterozygotes for a pathogenic variant and a modifier *trans* allele also show microtia (pedigree EUR01). This genotype combination is *FOXI3 Mut / FOXI3 modifier;* (iii) heterozygotes for a pathogenic variant and a non-modifier *trans* allele: there is no microtia in these cases; instead, the external ear could be normal or mildly dysmorphic. This genotype combination is *FOXI3 Mut / FOXI3 wild type or normal*. To test this hypothesis, we sequenced the genome of selected individuals, or genotyped selected polymorphic variants.

The sequence of the *FOXI3* deletion case EUR06 provided a candidate *trans FOXI3 modifier* haplotype. This 94.83 kb haplotype (from rs11686866 to rs111497808 including *FOXI3*) was present in heterozygosity in 7 of 25 (28%) CFM cases with types II or III microtia. There are two Linkage Disequilibrium (LD) blocks (A and B) in this haplotype (Fig. 4B). The A LD block extends for 25.24 kb from rs11686866 to rs66891658; the B LD block spans 59.1 kb 5′ to the *FOXI3* gene, from rs56297047 to rs111497808. A total of 21 cases with microtia have the A LD and/or B LD blocks in heterozygosity (10 cases with A + B, 2 with A, and 9 with B) suggesting that there are at least two different sites contributing to the phenotypic variation, while 7 cases did not have this haplotype (Fig. 4C). Furthermore, there were 16 cases from different pedigrees with a likely pathogenic *FOXI3* variant without the modifier haplotype in which there was no microtia. The *P* value of the *χ*^2^ test of this comparison is 2.8E-05 (Fig. 4D). Family-based transmission disequilibrium testing showed that the modifier haplotype is also significantly (*P* = 0.0325) associated with CFM phenotype. The alleles of the polymorphic sites for the A LD and the B LD blocks are listed in Supplementary Fig. 3.

The *trans FOXI3 modifier haplotype* is absent in the unaffected carrier parents and siblings of the Pakistani family. In addition, the *trans FOXI3 modifier haplotype* is not present in the unaffected carrier father and brothers of family EUR01, while the proband with the combination *FOXI3 p.Arg240Cys / FOXI3 modifier* has microtia. There was a similar configuration in family CHN03. In pedigree CHN01, the phenotypic expression is variable and only 4 individuals showed microtia type III, while the other carriers have milder manifestations of the external ear. Three of these 4 individuals have the *FOXI3 modifier* B LD block and one the A LD block. However, there are also individuals who carry the *FOXI3 modifier* haplotype without severe microtia. Thus, the presence of the proposed *FOXI3 modifier* haplotype(s) could not explain all the cases of incomplete penetrance. The affected individuals have the *FOXI3 modifier* haplotype, while the carrier asymptomatic mothers lack this haplotype.

## Discussion

We describe the identification of pathogenic variants in *FOXI3* that cause one form of CFM. Approximately 3.1% of 670 CFM cases are associated with pathogenic variants of *FOXI3*. There is a possible genotype–phenotype correlation; the majority of *FOXI3* pathogenic variants result in CFM with isolated type III microtia.

The FOXI3 protein belongs to the *forkhead* transcription factor family involved in cell fate decisions during embryonic development^34,35^. The *forkhead* domain proteins bind DNA as monomers, dimers, or in a complex with other proteins^36^. This family of proteins in humans contains 50 members classified into 19 subfamilies, one of which contains FOXI1, FOXI2, and FOXI3^34,37,38^. Pathogenic variants in several members of this gene family cause various Mendelian disorders^34,39^, and are also involved in cancer. Some of these include *FOXC1* and *FOXE3* in anterior segment dysgenesis syndrome type 3 and type 2, respectively, *FOXF1* CNV, SNV, and lncRNA in lethal lung developmental defects, *FOXG1* in an atypical Rett-like syndrome, *FOXJ1* in one form of Ciliary Dyskinesia syndrome, *FOXL2* in blepharophimosis-epicanthus inversus and ptosis syndrome, *FOXE1* in the Bamforth-Lazarus syndrome, *FOXP1* in intellectual disability with language impairment, *FOXP2* in speech and language disorder, *FOXN1* in T cell immunodeficiency, alopecia and nail dystrophy, and *FOXP3* in immune regulation polyendocrinopathy syndrome^34^. The gene *FOXI1*, the closest relative of *FOXI3* is involved in Pendred / Enlarged Vestibular Aqueduct syndrome, an autosomal recessive disorder characterized by the combination of goiter and inner ear anomalies^40^.

The molecular cause of the reduced penetrance is elusive and requires a better understanding of the regulation of *FOXI3* expression during development. Potential causes of the reduced penetrance include: (1) Stochastic monoallelic expression^41^ of the mutant allele. (2) The nature of the *trans FOXI3* allelic region. The hypothesis that a *trans* haplotype harbors a regulatory variant that modifies the expression of its *cis*-*FOXI3* gene is discussed in the “Results”, and has been described as a Compound Inheritance Gene Dosage model^42^. Examples include the Thrombocytopenia Absent Radius (TAR)^43^ and the *TBX6*-related congenital scoliosis^42^. (3) The presence of *cis* or *trans* eQTLs associated with the expression levels of *FOXI3*, as observed for *FOXF1* in a lethal lung developmental disorder^44^. The expression level of *FOXI3* is very low (https://gtexportal.org/home/ and ref. ^45^) and no eQTLs have been identified. (4) Digenic inheritance similar to some families with Retinitis Pigmentosa^46^ and Bardet-Biedl syndrome^47^. (5) Somatic mutations (a second hit hypothesis). This two-hit phenomenon is common in cancer^48^, and has been shown to occur in developmental disorders such as in Hereditary Hemorrhagic Telangiectasia^49^. (6) Epigenetic modifications other than imprinting as in the case of *DNMT3B* variants that modify epigenetic repression of the D4Z4 repeat and the penetrance of facioscapulohumeral dystrophy^50^. (7) Non-genetic factors including environmental effects.

The potential mechanism of the modifier *FOXI3* containing haplotype described here could be due to common variants of an enhancer sequence that regulate the level of *FOXI3* expression. One testable hypothesis is that the modifier haplotype is linked to a reduced expression of the normal *FOXI3* and therefore the effect of the mutant *FOXI3* allele is stronger. In contrast, all the other *FOXI3* haplotypes are linked to higher expression of the normal *FOXI3* gene and thus the effect of the mutant *FOXI3* is reduced or it is negligible. An analogous example is that of a hypomorphic allele of the *FECH* gene that increases the penetrance of the autosomal dominant erythropoietic protoporphyria when it occurs in *trans* to a pathogenic *FECH* variant^51^.

Heterozygous *FOXI3* mutations have been observed in some hairless dog strains; such dogs have not only hair defects but also dental defects. The *FOXI3* variant in hairless dogs is a dominant negative neomorphic mutation^52^. The hairless dog phenotype is always inherited in an autosomal semidominant fashion. The mutation in these dogs occurs early in the coding regions and produces a new protein as a result of a 7 bp duplication that shifts the reading frame. In two previous collaborative studies, conditional mouse knockouts of *Foxi3* were created in teeth and hair, and although tooth and hair defects were present in the conditional null mice, these defects were significantly less strong than those observed in *Foxi3* mutant dog breeds^53,54^.

The severity of phenotypic expression of a causative variant is related to the extensive genomic variation of each individual; the severity of sickle cell anemia in different populations provides an excellent example^55^. The possibility of imprinting of the *FOXI3* locus could be excluded based on the pedigrees studied. In addition, *FOXI3* has not been identified as an imprinted gene; however, transient or tissue-specific imprinting could not be excluded^56,57^.

The phenotypic expression of the pathogenic *FOXI3* variants is variable. This is evident in family CHN01, where the p.(Arg236Trp) results in various ear and facial phenotypes. Potential explanations may include: the random monoallelic expression of *FOXI3* during development^41^; the randomness of a developmental process when a key protein is dysfunctional or reduced as in Kartagener syndrome where heart looping is randomized between the left and right sides^58^. In almost all syndromes of abnormal development of fingers and toes, the phenotype varies between the two sides as for example in the *GLI3*-related Pallister-Hall syndrome^59^.

Previously reported *Foxi3*^*–/–*^ mice show abnormalities in the inner ear, the jaw, external, and middle ear, heart and artery development, and thymus^27–29^, while the severity of organs involved is quite different from CFM. However, our single-point mutation mice did not have visible malformations. This may be explained by genetic, developmental and physiological differences between mice and humans^60^, or the poor sequence homology (69.74%) between mice and humans for FOXI3; in addition, the timing of facial development and the rate of growth of embryonic facial precursors is quite different between mice and humans, which may underlie the differences in sensitivity to changes in the activity levels of transcription factors including FOXI3. However, the triple-mutation mice show many similar anomalies to human CFM phenotypes.

Different tissues in mice and humans have differing sensitivities to a reduction in Foxi3 function. *Foxi3* is expressed in the ectoderm and endoderm of the pharyngeal arches but not in the migrating crest cells that populate these arches^30^. The thymus, originating from the third pharyngeal pouch, is the most sensitive. Foxi3 heterozygous mice and patients with a loss of one allele of *FOXI3* show thymus defects and T-cell lymphopenia^61^. The patients described in ref. ^48^ all have large deletions that include the *FOXI3* gene, and one had craniofacial abnormalities. The external ear is the second most sensitive structure, with microtia associated with many of the human *FOXI3* variants. In the homozygous triple-mutant mice, microtia is fully penetrant, and is associated with perturbed development of the inner ear and middle ear ossicles, anomalies of the cranial base and palate, and syngnathia. The inner ear defects are derived from *Foxi3*-expressing preplacodal tissue^30,31,62^, while the middle ear and mandibular structures are derived from neural crest cells migrating into the first and second pharyngeal arches.

Studies on the gene network of FGF signaling (particularly FGF19) during otic induction in chick embryos have placed Foxi3 in the first step of otic induction i.e., the formation of the otic-epibranchial progenitors in the posterior pre-placodal region^63,64^. Additional proteins besides Foxi3, involved in this FGF signaling are Mapk, Etv5, Gbx2, Pax2, Six1, Six4, and Eya2^63,64^. Thus, all of these proteins could also be candidates for pathogenicity in CFM cases. Previously studies found that Foxi3-Gata3 and Foxi3-Fgf8 signaling is essential for facial morphogenesis^30,31,65^, and genome-wide association studies also found 15 CFM-related genes^15,16^. Variation in these genes could also be candidates of modifiers for the CFM phenotypes. However, we did not find any coding variants in them in the cohort of patients described here.

In conclusion, we provide genetic, genomic, and biological experimental evidence that likely pathogenic *FOXI3* variant alleles cause one form of Craniofacial Microsomia (CFM).

## Methods

### Ethics statement

All procedures involving human samples were performed with the approval of the Swiss Bioethics Committee of the University Hospitals and the Canton of Geneva (CER 11-036) and the Ethics Committees of the School of Biological Science and Medical Engineering, Beihang University (BM20210057), the Beijing Tongren Hospital, Capital Medical University, and the Plastic Surgery Hospital of the Peking Union Medical College and conducted under the Principles of Helsinki or an IRB approved protocol from USA. The study has been registered and approved by China’s Ministry of Science and Technology (project 2023-CJ0849). All participants or their guardians signed informed consent forms for biological investigation. All the European samples were collected after informed consent from the patients or their parents. Consent for the publication of photographs was given after a separate explanation and request. The FFF0062013 fibroblasts cell line was obtained from the Galliera Genetic Bank in Genova, Italy under the appropriate ethics approval for the distribution of samples (https://openbioresources.metajnl.com/articles/10.5334/ojb.15/). All animal experiments were performed under the approval of the Animal Care Committee at Beihang University (BM20210057) and the Institutional Animal Care and Use Committee at Baylor College of Medicine (D16-00475).

### Patient samples

The Chinese subjects were collected from the Beijing Tongren Hospital and the Plastic Surgery Hospital of the Peking Union Medical College. For the Chinese cohort, exome sequencing and target-capture sequencing were performed on 159 enrolled individuals from 48 CFM families and 498 sporadic CFM patients (Supplementary Table 2). The 48 probands include 36 males and 12 females and the 498 sporadic CFM patients include 396 males and 102 females. In the Pakistani family, ten individuals were enrolled including five males and five females. In the European patients, 124 probands (including 83 males and 41 females) were included in the project. The clinical characteristics of the Chinese CFM patients are shown in Supplementary Table 2. Ages range from 5 to 18 years in probands from CFM families and from 5 to 41 years in sporadic CFM patients. Clinical diagnosis for CFM is based on the OMENS classification system.

### Exome sequence and computational analyses

#### Exome sequencing

Genomic DNA from blood was extracted using standard procedures. DNA libraries for exome sequencing were prepared from 2 μg of qualified genomic DNA, which was sheared to 180–200 base pairs. The targeted coding regions of more than 20,000 genes were captured by Agilent SureSelect Human All Exon kit (V6) (5190-8883, Agilent Technologies, Inc., CA, USA) and Twist according to the manufacturer’s protocol. Paired-end sequencing (2 × 150 bp) was performed on the Illumina Xten or NovaSeq platforms (Illumina Inc., CA, USA) at Compass Biotechnology company (Whole-exome sequencing) or IGeneTech Biotechnology company (Target-capture sequencing) (Beijing, China).

### Dataset processing and family-based burden test

Burrows–Wheeler aligner tool (v0.7.17)^66^, SAMtools (v1.9)^66^, PICARD (v2.0.1), and the Genome Analysis Toolkit (v4)^67^ were used to analyze the exome/target-capture sequencing data. Sequenced reads were aligned to the GRCh38/hg38 reference human genome, and the filtering of variants was performed as described in previous studies^68–70^.

To prioritize the potential causal genes, family-based gene burden tests were performed on the rare loss-of-function variants of the 48 CFM families, and the promising variants were screened in the prioritized genes of each family according to inheritance patterns. The detailed steps were as follows: Firstly, the 273,164 high-quality variants were annotated by ANNOVAR software (Version: 2018Apr16)^71^, and the loss-of-function variants including splicing-site variants, missense variants, stop-gain- and stop-loss- variants, and frameshift variants were retained. Secondly, the variants with minor allele frequency less than 0.001 in the Genome Aggregation Database (gnomAD v3.1.2) were kept considering CFM is a rare congenital disorder. Thirdly, family-based burden tests were performed on the rare loss-of-function variants of the enrolled 48 families using Pedgene software (v3.6)^72^. The genes with burden *P*-values less than 0.001 were selected as candidates for subsequent analysis, and the detailed burden test statistics were shown in Supplementary Table 2. Fourthly, the variants within candidate genes were screened according to the inheritance pattern including autosomal dominant, autosomal recessive, de novo, or compound heterozygous. Fifthly, the effect of each variant identified from the above step was evaluated by SIFT (http://provean.jcvi.org/index.php), Polyphen-2 (http://genetics.bwh.harvard.edu/pph2/), and CADD (https://cadd.gs.washington.edu/) software. The variants predicted as “deleterious” or “probably damaging” or with CADD score larger than 10 were considered pathogenic ones. In addition, the functional effects on protein of the remained candidate single amino acid substitutions were predicted by SNAP2 software (v3.0, https://rostlab.org/services/snap2web/).

### In silico analysis

To assess the conservation of the mutated sites, multiple alignments were performed on sequences of FOXI3 among different species. Then the nuclear localization sequence (NLS) of FOXI3 was predicted by NucPred software (https://nucpred.bioinfo.se/cgi-bin/single.cgi) and the effect of the variants within NLS was evaluated by NLStradamus software (version: r.9, http://www.moseslab.csb.utoronto.ca/NLStradamus/) with default parameters.

To further predict the effect of the missense variants located in the conserved FHD or NLS on the three-dimensional (3D) structure, the sequence from 135 to 243 amino acid residues of human FOXI3 was primarily mapped to the other members of FOX family with known crystal structure referred to PDB database (https://www.rcsb.org/), including HNF-3 (1VTN), FOXA2 (5×07), FOXC2 (6AKO), Foxk1A (2C6Y), and FOXO1 (4LG0); and a phylogenetic tree of these proteins was then constructed by MEGA-X software (Version:10.0.5) (Supplementary Fig. 5A). Based on the phylogenetic tree and homologous evaluation, the crystal structures of 6AKO and 2C6Y were used to model the homologous sequence of FOXI3 by Modeller software (Version:9.25) (Supplementary Fig. 5B, 5C). The modeled 3D-structure of mutant proteins was presented through Mutagenesis function in UCSF Chimera software (Version:1.16) (Supplementary Fig. 5E). In addition, the crystal structures of the monomer and protein-DNA complex of 6AKO were used to mimic the effect of variants on DNA-binding for FOXI3 by FoldX software (Version:5.0). The prediction of free energy changes of each variant on monomer and protein-DNA complex was replicated 10 times with default parameters. The consequence of the variant on the protein was classified as “destabilize fold” when the difference of free energy (ΔΔ*G*) > 1.6 kcal/mol for the monomer protein between wild type and mutant. When the monomer ΔΔ*G* ≤ 1.6 kcal/mol, and the difference between the ΔΔG for the protein-DNA complex and the monomer >0.25 kcal/mol, the consequence of variants on the protein was classified as “perturb protein-DNA interaction” (Supplementary Fig. 5D).

### Sanger sequencing

Sanger sequencing was performed to validate or to discover variants in *FOXI3*. The primer design for the two exons of *FOXI3* was done using Primer3Web (Version: 4.1.0). PCR amplification of *FOXI3* exon 1 was performed on each sample in 50 µl reaction using 100 ng DNA, PCR primers (*FOXI3*_exon1_F1: ATGGAAATCCCAGCTCACAC; *FOXI3*_exon1_R1: TCATTCGTACTGTTCGGGTTA), the KAPA2G Robust HotStart PCR Kit (KK5522, Sigma-Aldrich, St. Louis, MO, USA) using KAPA Enhancer 1 and KAPA2G GC Buffer. The exon1 PCR reaction conditions were: 5 min at 95 °C, 40 cycles (15 s at 95 °C, 10 s at 60 °C, 3 min at 72 °C) and 2 min at 72 °C. PCR amplification of *FOXI3* exon 2 was performed on each sample in a 50 µl reaction using 50 ng DNA and the JumpStart™ REDTaq® DNA-Polymerase Kit (D8187-50UN, Merck, Kenilworth, NJ, USA) and PCR primers (*FOXI3*_exon2_F2: CAGGTGTGCATACTCAAGTCT; *FOXI3*_exon2_R2: AGGTCCTTCTCTAACTTGGTCTT). The exon 2 PCR reaction conditions were: 2 min at 94 °C, 35 cycles (60 s at 95 °C, 30 s at 58 °C, 5 min at 72 °C) and 2 min at 72 °C.

PCR was performed in 96-well plates (Biometra, Jena, Germany). Primers and dNTPs were digested prior to sequencing by enzymatic treatment using Shrimp Alkaline Phosphatase (M9910, Promega, WI, USA) and Exonuclease I (M0293, New England Biolabs, Ipswich, Madison, MA, USA). About 1.55 µl dH_2_O, 3U Exonuclease I and 6U SAP were added to 10 µl PCR product, incubated at 37 °C for 30 min, then at 80 °C for 15 min.

Sequencing reactions were conducted using Applied Biosystems Big Dye Terminator chemistry (4337455, Applied Biosystems, Carlsbad, CA, USA), and the products were resolved on the ABI Prism 3730XL DNA Analyzers (Applied Biosystems, Carlsbad, CA, USA). All the results were analyzed and visualized by Chromas software (Version: 2.6.5).

### Genome sequence

The genome sequence was performed in the accredited laboratory of Novagene (https://en.novogene.com/). A total of 0.5 μg of genomic DNA per sample was used for library preparation. Sequencing libraries were generated using the TruSeq Nano DNA HT Sample Prep Kit (20015965, Illumina Inc., CA, USA) following manufacturer’s recommendations. Genomic DNA was fragmented by sonication to a median size of 350 bp. Whole Genome Sequence libraries were sequenced on the Illumina HiSeq X TEN platform (2 × 150-bp paired-end reads) to a depth of 30X. Data were analyzed using the Saphetor computational platform (https://saphetor.com).

### Bionano optical mapping

Fibroblasts of the FFF006-2013 case^19^ have been obtained from the biorepository of the Istituto Giannina Gaslini (www.biobanknetwork.telethon.it) in Genova and cultured in RPMI 1640 (72400047, Gibco, Thermo Fisher, Paisley, Scotland, UK) supplemented with 2% L-Glutamine (25030149, Gibco, Thermo Fisher, Paisley, Scotland, UK) 15% fetal bovine serum (10100147, Gibco, Thermo Fisher, Paisley, Scotland, UK) and 1% penicillin/streptomycin/fungizone mix (Amimed, BioConcept, Allschwill, Switzerland) at 37 °C in a 5% CO_2_ atmosphere to reach 1.5 million cells. High molecular weight was prepared by the Bionano genomics (www.bionanogenomics.com). Labeling of the DNA was done using the DNA Bionano labeling kit; linearization and imaging were done using the manufacturer’s procedure of the Saphyr Chip in one of the flowcells. The computational analysis and visualization of the data were done with the Analysis Bionano Solve software (Version: 3.3.1).

### PacBio long-read sequence

PacBio circular consensus sequencing (CCS) was used for long-read sequencing targeted to the *FOXI3* locus in 20 CFM probands. Briefly, a ~7 kb region encompassing the entirety of the *FOXI3* locus was targeted for PCR amplification, followed by ligation to SMRTbell adapters to create circularized molecules from dsDNA. DNA polymerase and primers are annealed to circularized molecules to generate 1000 s of reads from a single molecule. Adapters are removed from reads to generate subreads, and subreads are used to discern consensus sequence of the region with >99% accuracy for further analysis. Bam files were viewed in IGV and cross-referenced with variant call files to analyze SNV and indel variants found by PacBio CCS. The 9 bp duplication found in BAB9863 from Baylor College of Medicine was Sanger sequenced for orthogonal validation. Cloning was performed using TOP10 competent cells and standard cloning procedure to isolate alleles and confirm in cis vs in trans inheritance of the indel variant with non-coding variants, as well as to confirm 9-bp in-frame duplication.

### Cell culture

HEK-293T cell line (1101HUM-PUMC000091) was obtained from the Cell Resource Center, Peking Union Medical College (which is the headquarter of National Science & Technology infrastructure—National BioMedical Cell-Line Resource, NSTI-BMCR). The HEK-293T cells were maintained in DMEM (Gibco Laboratories, NY, USA) supplemented with 10% FBS (Gibco Laboratories, NY, USA) in a humidified incubator at 37 °C with 5% CO2.

### Plasmid constructs

The entire coding sequence of *FOXI3* was cloned into the pIRES2-EGFP eukaryotic overexpression vector (GV146, Genechem Co., Ltd., Shanghai, China). The mutants of *FOXI3* were generated using the Q5 Site-Directed Mutagenesis Kit (E0554, New England BioLabs, Beverly, MA, USA) and verified by Sanger sequencing. The *AE4* promoter sequence was cloned into the luciferase vector (GV238, Genechem Co., Ltd., Shanghai, China). The wild type and mutant variants of *FOXI3* were cloned into the pEGFP-N1 vector (Clontech Laboratories, Inc., Palo Alto, CA, USA) to construct a C-terminus EGFP fused FOXI3 expression vector, respectively. Endotoxin-free plasmid was prepared using EndoFree Maxi Plasmid Kit (4992194, Tiangen, Shanghai, China) according to the manufacturer’s instructions.

### Luciferase assay

To perform luciferase assays, HEK-293T cells were seeded in 24-well plates and allowed to reach 80% confluence. Then, the cells were transfected with 0.25 μg of FOXI3 expression vectors, 0.25 μg of AE4 promoter vectors, and 0.01 μg of pRL-TK vectors using Lipofectamine 3000 transfection reagent (L3000008, Thermo Fisher, Paisley, Scotland, UK). Twenty-four hours after transfection, the cells were harvested and lysed, and the fluorescence intensity was measured by the Dual-Luciferase assay kit (E1910, Promega Corporation, Madison, WI, USA) according to the manufacturer’s instructions.

### Confocal analysis

The HEK-293T cells were cultured in 20 mm glass bottom confocal dishes (801001, Nest Scientific, Wuxi, Jiangsu, China) until they reached 80% confluence. Then, the cells were transfected with 1.5 μg FOXI3-EGFP or mutant FOXI3-EGFP expression vectors using Lipofectamine 3000 transfection reagent (L3000008, Thermo Fisher, Paisley, Scotland, UK) according to the transfection protocol. Twenty-four hours after transfection, the Hoechst 33342 (Beyotime Institute of Biotechnology, Haimen, China) was added to the medium. After incubation for 20 min, the cells were washed once with PBS. The high-speed confocal platform Dragonfly 200 (Andor Technology, Belfast, UK) and Fusion v2.3.0.36 (Andor Technology, Belfast, UK) were used to acquire and analyze the images.

### Western-blot assay

Twenty-four hours after transfection, the HEK-293T cells were harvested to separate the proteins into cytoplasmic and nuclear proteins by the Nuclear and Cytoplasmic Protein Extraction Kit (P0027, Beyotime Institute of Biotechnology, Haimen, China) according to the manufacturer’s instructions. The protein concentrations of the cytoplasmic and nuclear constituents were determined by the BCA Protein Assay Kit (P0010, Beyotime Institute of Biotechnology, Haimen, China). The cytoplasmic and nuclear proteins were then separated by 4–20% Tris-Gly gel (GSG2001-420F, WSHT, Shanghai, China) and transferred to PVDF membranes (IPVH0010, Millipore, Billerica, MA, USA), respectively. After blocking with 5% w/v nonfat milk, the membranes were incubated with specific primary antibodies overnight at 4 °C. Subsequently, the membranes were incubated with HRP-conjugated secondary antibodies at room temperature for 1 h. Blots were visualized by chemiluminescence reagent (WBKLS0100, Millipore, Billerica, MA, USA). The primary antibodies is anti-EGFP (ab184601, Abcam, Cambridge, UK; 1:1000 dilution), and secondary antibody is Goat ani mouse lgG(H + L)-HRP (P03S01L, Gene-Protein Link Biotech,Beijing, China; 1:10,000 dilution); nuclear protein internal control is anti-H3 (ab1791, Abcam, Cambridge, UK; 1:5000 dilution) and secondary antibody is Goat anti-Rabbit lgG(H + L)-HRP (P03S02L, Gene-Protein Link Biotech,Beijing, China ;1:10,000 dilution); cytoplasmic protein internal control is anti-GAPDH (ab8245, Abcam, Cambridge, UK; 1:5000 dilution) and secondary antibody is Goat ani mouse lgG(H + L)-HRP (P03S01L, Gene-Protein Link Biotech,Beijing, China ;1:10,000 dilution). The band density was quantified by ImageJ software v1.8.0.345 (National Institutes of Health, Bethesda, Maryland, USA). The uncropped and unprocessed scans of the western blots were provided in the Source Data file.

### Constructing *Foxi3* variant mouse models using CRISPR

#### Generation of triple variant mice

C57BL/6 mice carrying the three variants p.(Arg220Trp), p.(Arg222Gln), and p.(Arg224His), orthologous to FOXI3:p.(Arg236Trp), p.(Arg238Gln) and p.(Arg240His) in one allele were generated by Cas9/CRISPR-mediated genome editing (Cyagen Biosciences, Guangzhou, China). Briefly, a gRNA (TCGGAAGCGAAGGCGCCGCG-CGG) to the mouse *Foxi3* gene, a donor oligo with a targeting sequence containing p.(Arg220Trp) (codon from CGG to TGG), p.(Arg222Gln) (codon from CGA to CAA) and p.(Arg224His) (codon from CGC to CAC) mutations sites, and Cas9 protein were co-injected into fertilized mouse eggs to generate targeted knock-in offspring. The genotype of pups was determined by Sanger sequencing using 5′-CATGCACTAAAGTGTCTTGGAACAT-3′ and 5′-TTAAAGGTGCTGAGGAAAGTGTTG-3′ as PCR primer and 5′-GTTGAGACACGGAGTGGAGG-3′ as sequencing primer. Heterozygous mice *Foxi3*^*R220W-R222Q-R224H/+*^ were mated with *Foxi3*^*+/+*^ C57BL/6 mice to acquire heterozygous progenies, which were then intercrossed to generate homozygous *Foxi3*^*R220W-R222Q-R224H/R220W-R222Q-R224H*^ mice.

#### Generation of *Foxi3*^*R224H/+*^ mice

The generation and genotyping of *Foxi3*^*R224H/+*^ mice were consistent with the above methods except for the donor oligonucleotide containing the variant of p.(Arg224His) (codon from CGC to CAC).

#### Generation of *Foxi3*^*F218L/+*^ mice

C57BL/6 mice carrying the variant p.(Phe218Leu), orthologous to *FOXI3*:p.(Phe233Leu) were generated by Cas9/CRISPR-mediated genome editing. A gRNA (CGCGGCGCCTTCGCTTCCGA-CGG) to the mouse Foxi3 gene, a 135 bp donor oligonucleotide flanking the targeted site and containing p.(Phe218Leu) (codon from TTC to CTG), and Cas9 protein were co-injected into fertilized mouse eggs to generate targeted knock-in offspring. Correct targeting and absence of indels were verified by Sanger sequencing of exon 2 of *Foxi3* containing the modified allele. Genotyping was performed using 5′-CAGAGAAACCCTGCCTCGAA-3′ and 5′-AGGAGGCAGTACTCTTGGTG-3′ to generate a 368 bp product. The presence of the modified *Foxi3* allele was revealed by digesting the amplified PCR band with MwoI to produce two bands of 187 and 181 bp; the wild-type band was not cut by MwoI and ran at 368 bp. Heterozygous *Foxi3*^*F218L/+*^ mice were crossed with wild type mice for several generations, and the heterozygous offspring intercrossed to generate homozygous *Foxi3*^*F218L/F218L*^ mice. Homozygous mice were mated with *Foxi3* null mice^20,22^ (*Foxi3*^*+/-*^) to generate *Foxi3*^*F218L/-*^ mice.

All animals used in this study were bred and maintained under specific pathogen-free conditions with a temperature of 18–24 °C and humidity of 35–60% and 12-h light/dark cycle and free access to food and water.

### Phenotypic analysis of mouse models

#### Skeletal staining

Newborn mice were euthanized, stored in PBS on ice, and then scalded the fetuses in hot tap water (70 °C) for approximately 15 s. The skin was carefully peeled off and all the internal organs were cut away with forceps. The bodies were fixed in 95% ethanol for 24 h and stained with Alizarin Red and Alcian Blue as described in previous studies^73^ and photographed with bright field microscopy.

### Micro-computed tomography (Micro-CT) analysis of skull tissue

Newborn mice were euthanized, fixed in 4% PFA and scanned with a photon counting detector-based spectral Micro-CT developed by the Institute of High Energy Physics, Chinese Academy of Sciences. The parameters are used as follows: 20 μm resolution, 50kVp, 144mAs without a filter. Raw images from all scans were reconstructed using the FDK algorithm. Reconstructed scan data were imported into VG Studio software version 3.1(Volume Graphics, Inc., NC, USA) and Drishti Volume Exploration Software^74^ for 3D rendering of bone and soft tissue.

### Reporting summary

Further information on research design is available in the Nature Portfolio Reporting Summary linked to this article.

## Supplementary information

Supplementary Information

Reporting Summary

## Data Availability

The likely pathogenic *FOXI3* variants described in this study have been submitted to the ClinVar database (https://www.ncbi.nlm.nih.gov/clinvar/) and their accession numbers are SCV003803092-SCV003803100 and SCV003806728-SCV003806732. The raw datasets (FASTQ files) supporting this study have not been deposited in a public repository because of confidentiality of medical tests; however, VCF files for selected genes are available under restricted access from S.E.A. (stylianos.antonarakis@unige.ch) or Y.-B.Z. (zhangyongbiao@buaa.edu.cn) upon request. The access to these data requires a short description of the project, and signing a data-use agreement that includes restrictions on downstream data, and use of the data only by the requesting investigator. There is no authorship requirement for a reanalysis of the data unless there is an extensive follow-up collaboration and input from the authors of this paper. The response timeframe will be less than 3 weeks. The mouse models described here are also available upon request from Y.-B.Z. (zhangyongbiao@buaa.edu.cn) or A.K.G. (akgroves@bcm.edu). Frequency data of selected *FOXI3* variants in different human populations have been obtained from gnomAD (https://gnomad.broadinstitute.org/). Additional datasets required to reproduce the analysis and figures are in Source data. Source data are provided with this paper. GRCh38/hg38 reference human genome (https://hgdownload.soe.ucsc.edu/goldenPath/hs1/vsHg38/); Publicly available protein structures were obtained from the Protein Data Bank (https://www.rcsb.org/). All data supporting the findings described in this manuscript are available in the article and its Supplementary Information files, and from the corresponding authors upon request. Source data are provided with this paper.

## Source data

Source Data

## References

[1] Klein D (1990). Living history–autobiography: genetics and environment from a personal perspective. Am. J. Med. Genet..

[2] Goldenhar M (1952). Associations malformatives de l’oeil et de l’oreille, en particulier le svndrome dermoide epibulbaire- appendices auriculaires-fistula auris congenita et ses relations avec la dysostose mandibulo-faciale. J. Genet. Hum..

[3] Cohen MM, Rollnick BR, Kaye CI (1989). Oculoauriculovertebral spectrum: an updated critique. Cleft Palate J..

[4] Beleza-Meireles A, Clayton-Smith J, Saraiva JM, Tassabehji M (2014). Oculo-auriculo-vertebral spectrum: a review of the literature and genetic update. J. Med. Genet..

[5] Mastroiacovo P (1995). Epidemiology and genetics of microtia-anotia: a registry based study on over one million births. J. Med. Genet..

[6] Shirazi M, Abbariki E, Pirjani R, Akhavan S, Dastgerdy E (2015). Congenital microtia in a neonate due to maternal isotretinoin exposure 1 month before pregnancy: case report. J. Obstet. Gynaecol. Res..

[7] Adam AP (2020). Recurrent constellations of embryonic malformations re-conceptualized as an overlapping group of disorders with shared pathogenesis. Am. J. Med. Genet. A.

[8] Artunduaga MA (2009). A classic twin study of external ear malformations, including microtia. N. Engl. J. Med..

[9] Tingaud-Sequeira A (2021). A recurrent missense variant in EYA3 gene is associated with oculo-auriculo-vertebral spectrum. Hum. Genet..

[10] Lopez E (2016). Mutations in MYT1, encoding the myelin transcription factor 1, are a rare cause of OAVS. J. Med. Genet..

[11] Tingaud-Sequeira A (2020). Functional and genetic analyses of ZYG11B provide evidences for its involvement in OAVS. Mol. Genet. Genom. Med..

[12] Rengasamy Venugopalan S (2019). A novel nonsense substitution identified in the AMIGO2 gene in an Occulo-Auriculo-Vertebral spectrum patient. Orthod. Craniofac. Res..

[13] Wang Y (2020). A mutation in VWA1, encoding von willebrand factor a domain-containing protein 1, is associated with hemifacial microsomia. Front. Cell Dev. Biol..

[14] Timberlake AT (2021). Haploinsufficiency of SF3B2 causes craniofacial microsomia. Nat. Commun..

[15] Xu X (2021). Novel risk factors for craniofacial microsomia and assessment of their utility in clinic diagnosis. Hum. Mol. Genet..

[16] Zhang YB (2016). Genome-wide association study identifies multiple susceptibility loci for craniofacial microsomia. Nat. Commun..

[17] Quiat D (2022). An ancient founder mutation located between ROBO1 and ROBO2 is responsible for increased microtia risk in Amerindigenous populations. Proc. Natl Acad. Sci. USA.

[18] Ansar M (2018). Bi-allelic loss-of-function variants in DNMBP cause infantile cataracts. Am. J. Hum. Genet..

[19] Tassano E (2015). Congenital aural atresia associated with agenesis of internal carotid artery in a girl with a FOXI3 deletion. Am. J. Med. Genet. A.

[20] Gutierrez-Arcelus M (2013). Passive and active DNA methylation and the interplay with genetic variation in gene regulation. Elife.

[21] Lappalainen T (2013). Transcriptome and genome sequencing uncovers functional variation in humans. Nature.

[22] Consortium GT (2017). Genetic effects on gene expression across human tissues. Nature.

[23] Taliun D (2021). Sequencing of 53,831 diverse genomes from the NHLBI TOPMed Program. Nature.

[24] Chen X (2019). Structural basis for DNA recognition by FOXC2. Nucleic Acids Res..

[25] Tsai KL (2006). Crystal structure of the human FOXK1a-DNA complex and its implications on the diverse binding specificity of winged helix/forkhead proteins. J. Biol. Chem..

[26] Schymkowitz J (2005). The FoldX web server: an online force field. Nucleic Acids Res..

[27] Youssoufian H (1986). Recurrent mutations in haemophilia A give evidence for CpG mutation hotspots. Nature.

[28] Kurth I (2006). The forkhead transcription factor Foxi1 directly activates the AE4 promoter. Biochem J..

[29] Singh S, Jangid RK, Crowder A, Groves AK (2018). Foxi3 transcription factor activity is mediated by a C-terminal transactivation domain and regulated by the protein phosphatase 2A (PP2A) complex. Sci. Rep..

[30] Edlund RK, Ohyama T, Kantarci H, Riley BB, Groves AK (2014). Foxi transcription factors promote pharyngeal arch development by regulating formation of FGF signaling centers. Dev. Biol..

[31] Birol O (2016). The mouse Foxi3 transcription factor is necessary for the development of posterior placodes. Dev. Biol..

[32] Hasten E, Morrow BE (2019). Tbx1 and Foxi3 genetically interact in the pharyngeal pouch endoderm in a mouse model for 22q11.2 deletion syndrome. PLoS Genet..

[33] Rieder MJ (2012). A human homeotic transformation resulting from mutations in PLCB4 and GNAI3 causes auriculocondylar syndrome. Am. J. Hum. Genet..

[34] Herman L, Todeschini AL, Veitia RA (2021). Forkhead transcription factors in health and disease. Trends Genet..

[35] Golson ML, Kaestner KH (2016). Fox transcription factors: from development to disease. Development.

[36] Dai S, Qu L, Li J, Chen Y (2021). Toward a mechanistic understanding of DNA binding by forkhead transcription factors and its perturbation by pathogenic mutations. Nucleic Acids Res..

[37] Hannenhalli S, Kaestner KH (2009). The evolution of Fox genes and their role in development and disease. Nat. Rev. Genet.

[38] Jackson BC, Carpenter C, Nebert DW, Vasiliou V (2010). Update of human and mouse forkhead box (FOX) gene families. Hum. Genom..

[39] Benayoun BA, Caburet S, Veitia RA (2011). Forkhead transcription factors: key players in health and disease. Trends Genet..

[40] Yang T (2007). Transcriptional control of SLC26A4 is involved in Pendred syndrome and nonsyndromic enlargement of vestibular aqueduct (DFNB4). Am. J. Hum. Genet..

[41] Borel C (2015). Biased allelic expression in human primary fibroblast single cells. Am. J. Hum. Genet..

[42] Wu N (2015). TBX6 null variants and a common hypomorphic allele in congenital scoliosis. N. Engl. J. Med..

[43] Albers CA (2012). Compound inheritance of a low-frequency regulatory SNP and a rare null mutation in exon-junction complex subunit RBM8A causes TAR syndrome. Nat. Genet..

[44] Szafranski P (2013). Small noncoding differentially methylated copy-number variants, including lncRNA genes, cause a lethal lung developmental disorder. Genome Res..

[45] Consortium GT (2020). The GTEx Consortium atlas of genetic regulatory effects across human tissues. Science.

[46] Kajiwara K, Berson EL, Dryja TP (1994). Digenic retinitis pigmentosa due to mutations at the unlinked peripherin/RDS and ROM1 loci. Science.

[47] Katsanis N (2001). Triallelic inheritance in Bardet-Biedl syndrome, a Mendelian recessive disorder. Science.

[48] Cavenee WK (1983). Expression of recessive alleles by chromosomal mechanisms in retinoblastoma. Nature.

[49] Snellings DA (2019). Somatic mutations in vascular malformations of hereditary hemorrhagic telangiectasia result in bi-allelic loss of ENG or ACVRL1. Am. J. Hum. Genet..

[50] van den Boogaard ML (2016). Mutations in DNMT3B modify epigenetic repression of the D4Z4 repeat and the penetrance of facioscapulohumeral dystrophy. Am. J. Hum. Genet..

[51] Gouya L (2002). The penetrance of dominant erythropoietic protoporphyria is modulated by expression of wildtype FECH. Nat. Genet..

[52] Drogemuller C (2008). A mutation in hairless dogs implicates FOXI3 in ectodermal development. Science.

[53] Jussila M (2015). Suppression of epithelial differentiation by Foxi3 is essential for molar crown patterning. Development.

[54] Shirokova V (2016). Foxi3 deficiency compromises hair follicle stem cell specification and activation. Stem Cells.

[55] Nagel RL (1985). Hematologically and genetically distinct forms of sickle cell anemia in Africa. The Senegal type and the Benin type. N. Engl. J. Med..

[56] Monk D, Mackay DJG, Eggermann T, Maher ER, Riccio A (2019). Genomic imprinting disorders: lessons on how genome, epigenome and environment interact. Nat. Rev. Genet..

[57] Santoni FA (2017). Detection of imprinted genes by single-cell allele-specific gene expression. Am. J. Hum. Genet..

[58] Afzelius BA (1976). A human syndrome caused by immotile cilia. Science.

[59] Al-Qattan MM, Shamseldin HE, Salih MA, Alkuraya FS (2017). GLI3-related polydactyly: a review. Clin. Genet..

[60] Cacheiro P, Haendel MA, Smedley D (2019). International mouse phenotyping, C. & the Monarch, I. New models for human disease from the International Mouse Phenotyping Consortium. Mamm. Genome.

[61] Bernstock JD (2020). Recurrent microdeletions at chromosome 2p11.2 are associated with thymic hypoplasia and features resembling DiGeorge syndrome. J. Allergy Clin. Immunol..

[62] Khatri SB, Groves AK (2013). Expression of the Foxi2 and Foxi3 transcription factors during development of chicken sensory placodes and pharyngeal arches. Gene Expr. Patterns.

[63] Tambalo M, Anwar M, Ahmed M, Streit A (2020). Enhancer activation by FGF signalling during otic induction. Dev. Biol..

[64] Anwar M, Tambalo M, Ranganathan R, Grocott T, Streit A (2017). A gene network regulated by FGF signalling during ear development. Sci. Rep..

[65] Abe M (2021). GATA3 is essential for separating patterning domains during facial morphogenesis. Development.

[66] Li H, Durbin R (2009). Fast and accurate short read alignment with Burrows–Wheeler transform. Bioinformatics.

[67] DePristo MA (2011). A framework for variation discovery and genotyping using next-generation DNA sequencing data. Nat. Genet..

[68] Makrythanasis P (2014). Diagnostic exome sequencing to elucidate the genetic basis of likely recessive disorders in consanguineous families. Hum. Mutat..

[69] Ansar M (2018). Visual impairment and progressive phthisis bulbi caused by recessive pathogenic variant in MARK3. Hum. Mol. Genet..

[70] Ansar M (2018). Biallelic variants in LINGO1 are associated with autosomal recessive intellectual disability, microcephaly, speech and motor delay. Genet. Med..

[71] Wang K, Li M, Hakonarson H (2010). ANNOVAR: functional annotation of genetic variants from high-throughput sequencing data. Nucleic Acids Res..

[72] Schaid DJ, McDonnell SK, Sinnwell JP, Thibodeau SN (2013). Multiple genetic variant association testing by collapsing and kernel methods with pedigree or population structured data. Genet. Epidemiol..

[73] Ovchinnikov D (2009). Alcian blue/alizarin red staining of cartilage and bone in mouse. Cold Spring Harb. Protoc..

[74] Hu Y, Limaye A, Lu J (2020). Three-dimensional segmentation of computed tomography data using Drishti Paint: new tools and developments. R. Soc. Open Sci..

