# Supplementary material for: FOXI3 pathogenic variants cause one form of craniofacial microsomia
Source: Nat Commun. 2023 Apr 11;14:2026. doi: 10.1038/s41467-023-37703-6 (PMC10090152; doi:10.1038/s41467-023-37703-6)
Supplement: Supplementary file 1 — Supplementary Information [file 41467_2023_37703_MOESM1_ESM.pdf]

Supplementary Information

FOXI3 pathogenic variants cause one form of craniofacial microsomia

Mao et al.

Supplementary Information contains

Supplementary Tables
- Supplementary Table 1
- Supplementary Table 2
- Supplementary Table 3
- Supplementary Table 4

Supplementary Figures
- Supplementary Figure 1
- Supplementary Figure 2
- Supplementary Figure 3
- Supplementary Figure 4
- Supplementary Figure 5
- Supplementary Figure 6
- Supplementary Figure 7
- Supplementary Figure 8
- Supplementary Figure 9
- Supplementary Figure 10
- Supplementary Figure 11
- Supplementary Figure 12

Supplementary References

Supplementary Tables

Supplementary Table 1. Summary of CFM pedigrees and patients with *FOXI3* variants

Family ID	Individual	Variants in *FOXI3*	Genotype	Presumed *FOXI3* *trans* Haplotype		Microtia Type	Microtia-related Phenotype
				A LD block (from rs11686866 to rs66891658)	B LD block (from rs56297047 to rs111497808)		
F252	IV : 1	p.(Phe234Leu)	homozygous	absent		Type III	Bilateral type III microtia and deafness
	IV : 5	p.(Phe234Leu)	homozygous	absent		Type III	Bilateral type III microtia and deafness
CHN01	II : 8	p.(Arg236Trp)	heterozygous	absent	B LD block	Type III	Unilateral (right) microtia III and atresia of the external auditory canal
	III : 1	p.(Arg236Trp)	heterozygous	absent	B LD block	Type III	Bilateral type III microtia, mild bilateral eyelid ptosis, severe micrognathia, irregular teeth arrangement, yellow-white mass can be seen in the temporal bulbar conjunctiva of the right eye
	III : 10	p.(Arg236Trp)	heterozygous	A LD block	B LD block	Type II	Unilateral (left) microtia II with shell-shaped ear, absence of the superior anti-helical crus, the external auditory canal was atretic
	III : 12	p.(Arg236Trp)	heterozygous	absent		Type I	Underdeveloped tragus, bilateral
	III : 13	p.(Arg236Trp)	heterozygous	A LD block	B LD block	Type III	No photos, described by his family as with unilateral (right) microtia III
	IV : 1	p.(Arg236Trp)	heterozygous	absent	B LD block	Type III	Unilateral (left) microtia III
	IV : 8	p.(Arg236Trp)	heterozygous	A LD block	absent	Type III	Bilateral type III microtia and atresia of the external auditory canals
	IV : 11	p.(Arg236Trp)	heterozygous	absent	B LD block	/	Abnormality in the posterior crus of the antihelix of the right ear
EUR01	EUR01	p.(Arg240Cys)	heterozygous	A LD block	B LD block	Type III	Unilateral (right side) type III microtia; Variant inherited from unaffected father, and present in two unaffected siblings

EUR02	EUR02	p.(Phe234Val)	heterozygous	A LD block	B LD block	Type III	Bilateral type III microtia; Variant inherited from affected father
EUR03	EUR03	p.(Cys225Arg)	heterozygous	absent	B LD block	Type III	type III microtia; Variant inherited from unaffected father
EUR04	EUR04	p.(Ala32GlyfsTer147)	heterozygous	absent	B LD block	Type III	Microtia type III; Variant inherited from unaffected mother
EUR05	EUR05	p.(Phe102Tyr)	heterozygous	absent	absent	Type III	Microtia type III
EUR06	EUR06	chr2:87317229-89306982 deletion	heterozygous	A LD block	B LD block	Type III	Unilateral type III microtia
CHN02	II : 1	p.(Arg238Glu)	heterozygous	absent	B LD block	Type III	Unilateral (right) type III microtia
CHN03	II : 1	p.(Arg240Cys)	heterozygous	absent	B LD block	Type III	Unilateral (right) type III microtia
CHN04	II : 1	p.(Arg240His)	heterozygous	absent		Type III	Unilateral (right) type III microtia
CHN05	II : 1	p.(Leu199Phe)	heterozygous	absent		Type III	Bilateral type III microtia
CHN05	II : 2	p.(Leu199Phe)	heterozygous	absent		Type III	unilateral (left) type III microtia
CHN-S001	CHN-S001	p.(Ser119_Ala124del)	heterozygous	A LD block	B LD block	Type III	Unilateral (left side) microtia type III
CHN-S002	CHN-S002	p.(Pro147Thr)	heterozygous	A LD block	absent	Type III	Unilateral (right side) microtia type III
CHN-S003	CHN-S003	p.(Ser169Asn)	heterozygous	absent		Type II	Unilateral (left side) microtia type II
CHN-S004	CHN-S004	p.(Arg235Cys)	heterozygous	A LD block	B LD block	Type III	Unilateral (left side) microtia type III
CHN-S005	CHN-S005	p.(Arg236Gln)	heterozygous	absent	B LD block	Type III	Bilateral type III microtia
CHN-S006	CHN-S006	p.(Arg238Gln)	heterozygous	A LD block	B LD block	Type III	Bilateral type III microtia
CHN-S007	CHN-S007	p.(Pro355Leu)	heterozygous	absent	B LD block	Type III	Unilateral (right side) type III microtia
CHN-S008	CHN-S008	p.(Ser373_Thr376del)	heterozygous	absent		Type III	Unilateral (right side) type III microtia

CHN-S009	CHN-S009	p.(Ser373_Thr376del)	heterozygous	absent		Type III	Bilateral microtia (left side, type I microtia; right, type III microtia, with appendage)
CHN-S010	CHN-S010	p.(Arg415Ter)	heterozygous	A LD block	B LD block	Type II	Left type II microtia, with appendage on the right side

Supplementary Table 2. Clinical characteristics of the Chinese CFM Patients

Variables	Probands (Value) (Chinese Families)	Probands (Value) (Chinese sporadic)
**No.**	48	498
**Age, year**		
Mean	8.7	11.2
Range	5-18	5-41
**Gender**		
Male	36 (75.0%)	396 (79.5%)
Female	12 (25.0%)	102 (20.5%)
**Laterality**		
Left	9 (18.8%)	187 (37.6%)
Right	37 (77.1%)	280 (56.2%)
Bilateral[a]	2 (4.2%)	31 (6.2%)
**Nagata definition**		
Anotia	0	9 (1.8%)
Lobule type	43 (89.6%)	372 (74.7%)
Small conchal type	1 (2.1%)	63 (12.7%)
Conchal type	4 (8.3%)	54 (10.8%)
**External auditory meatus of affected-side**		
Atresia	44 (91.7%)	386 (77.5%)
Stricture	4 (8.3%)	112 (22.5%)
**Temporal bone agenesis of affected-side**		
No	43 (89.6%)	383 (76.9%)
Yes	5 (10.4%)	115 (23.1%)
**Other craniofacial abnormal**		
Micromandible	1 (2.1%)	5 (1%)
Teeth irregular	3 (6.25%)	33 (6.7%)
**OMENS[b] classification**		
O0	19 (39.6%)	369 (74.1%)
O1	19 (39.6%)	80 (16.1%)
O2	4 (8.3%)	35 (7.0%)
O3	6 (12.5%)	14 (2.8%)
M1	24 (50.0%)	261 (52.4%)
M2A	15 (31.3%)	189 (38.0%)
M2B	9 (18.8%)	48 (9.6%)
E1	0	2 (0.4%)
E2	40 (83.3%)	60 (12.0%)
E3	8 (16.7%)	436 (87.6%)
S1	18 (37.5%)	331 (66.5%)
S2	22 (45.8%)	148 (29.7%)
S3	8 (16.7%)	19 (3.8%)

a Patients with Bilateral laterality were classified according to the more severe ears.
b OMENS, Orbit, Mandible, Ear, Nerve, and Soft tissue.

Supplementary Table 3. Burden test on rare loss-of-function variants of 48 Chinese CFM families

Gene	Chromosome	Number	stat.kernel	*P*-val.kernel	stat.burden	*P*-val.burden
FOXI3	2	6	6.55E+03	7.85E-08	5.31241	1.08E-07
OR8G1	11	5	1.27E+04	4.34E-05	4.13863	3.49E-05
WDR76	15	2	2.46E+03	8.15E-04	3.84219	1.22E-04
CEP112	17	3	1.57E+03	2.75E-03	3.75836	1.71E-04
OR8G5	11	1	1.40E+01	1.84E-04	3.74059	1.84E-04
OR8J3	11	1	1.40E+01	1.84E-04	3.74059	1.84E-04
TGOLN2	2	3	4.43E+03	5.85E-06	3.68044	2.33E-04
KIAA1211L	2	2	1.92E+03	3.20E-03	3.46858	5.23E-04
ZDHHC11B	5	6	1.54E+03	2.89E-03	3.43702	5.88E-04
CPSF3	2	1	1.16E+01	6.73E-04	3.4004	6.73E-04
DVL1	1	2	1.53E+03	2.91E-03	3.34116	8.34E-04
COL4A3	2	1	1.12E+01	8.37E-04	3.34036	8.37E-04
FEM1B	15	1	1.12E+01	8.37E-04	3.34036	8.37E-04
GART	21	1	1.12E+01	8.37E-04	3.34036	8.37E-04
OR2L3	1	1	1.12E+01	8.37E-04	3.34036	8.37E-04
TAF5	10	1	1.12E+01	8.37E-04	3.34036	8.37E-04

The table shows the output for top 16 associatied genes for CFM disease based on the 48 Chinese pedigree data. Number indicates the number of variants used, the non-informative variants that were removed. For the kernel statistic and *P*-value (without multiple adjusting; Gene with *P*-value less than 2.5E-06 (0.05/20,000) is considered as gene-wide significant), and the burden statistic and *P*-value (without multiple adjusting; Gene with *P*-value less than 2.5E-06 (0.05/20,000) is considered as gene-wide significant), the kernel *P*-value is calculated using Kounen's saddlepoint method[1], and the burden *P*-value is based on the normal distribution.

Supplementary Table 4. Exome and whole genome sequencing identified known candidate genes related to syndromes with microtia

Patient ID	Gene	Transcript	cHGVS	pHGVS	Consequence	Inheritance	Phenotype
CHN20	*CHD7*	NM_017780.3	c.G1779C	p.(Gln593His)	missense	compound heterozygous inheritance, inherited from unaffected father	Unilateral (right); microtia type III
			c.G3218A	p.(Gly1073Glu)	missense	compound heterozygous inheritance, inherited from unaffected mother	
CHN43	*CHD7*	NM_017780.3	c.G4516A	p.(Gly1506Ser)	missense	inherited from her unaffected father	Unilateral (right); microtia type II
CHN11	*TBX1*	NM_001330677.3	c.C491T	p.(Pro164Leu)	missense	inherited from her unaffected mother	Unilateral (right); microtia type III
CHN47	*TBX1*	NM_001330677.3	c.A1469G	p.(Try490Cys)	missense	inherited from his unaffected mother	Unilateral (right); microtia type III
CHN29	*SF3B2*	NM_006842.3	c.G784T	p.(Asp262Tyr)	missense	denovo	Unilateral (right); microtia type III
WGS02	*SF3B2*	NM_006842.3	c.G2542A	p.(Val848Met)	missense	unknown	Unilateral (left); microtia type III
CHN35	*DHODH*	NM_001361.5	c.610del	p.(Leu204TrpfsTer7)	frameshift	denovo	Unilateral (right); microtia type III
CHN07	*FGFR2*	NM_000141.3	c.A1213G	p.(Lys405Glu)	missense	inherited from her unaffected mother	Unilateral (right); microtia type III; appendage
CHN08	*EYA1*	NM_000503.3	c.G679A	p.(Ala227Thr)	missense	inherited from his unaffected father	Unilateral (left); microtia type III;

SUPPLEMENTARY FIGURES

Supplementary Figure 1

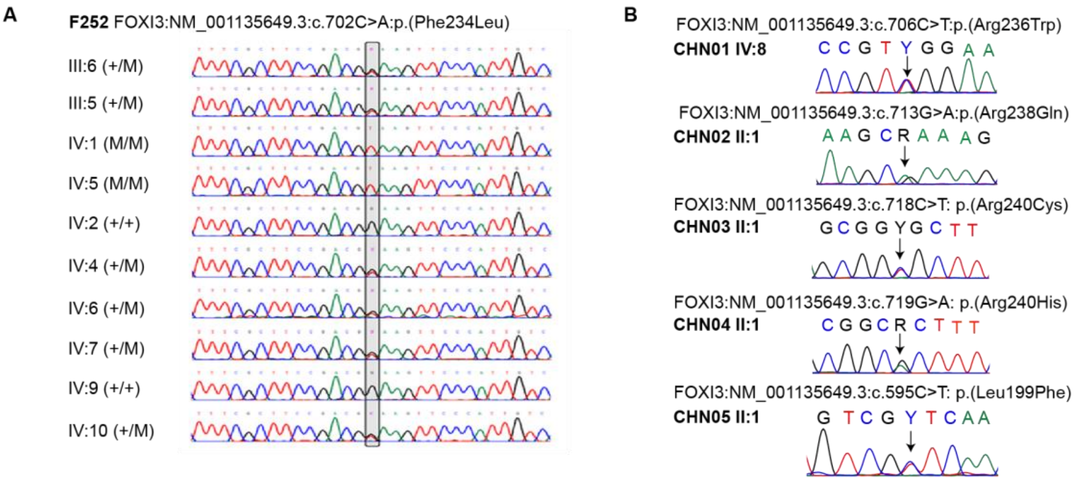

Supplementary Figure 1. Sanger sequencing validate variants identified from F252 and CHN01 families. A: The Sanger sequences of the likely pathogenic variant of *FOXI3*:c.702C>A for Pakistani family. The grey-shaded site shows the opposite strand sequence, that is *FOXI3*:c.702G>T. B: The Sanger sequences of the likely pathogenic variants identified from Chinese CFM families. The black arrow indicates the changes of the nucleotide.

Supplementary Figure 2

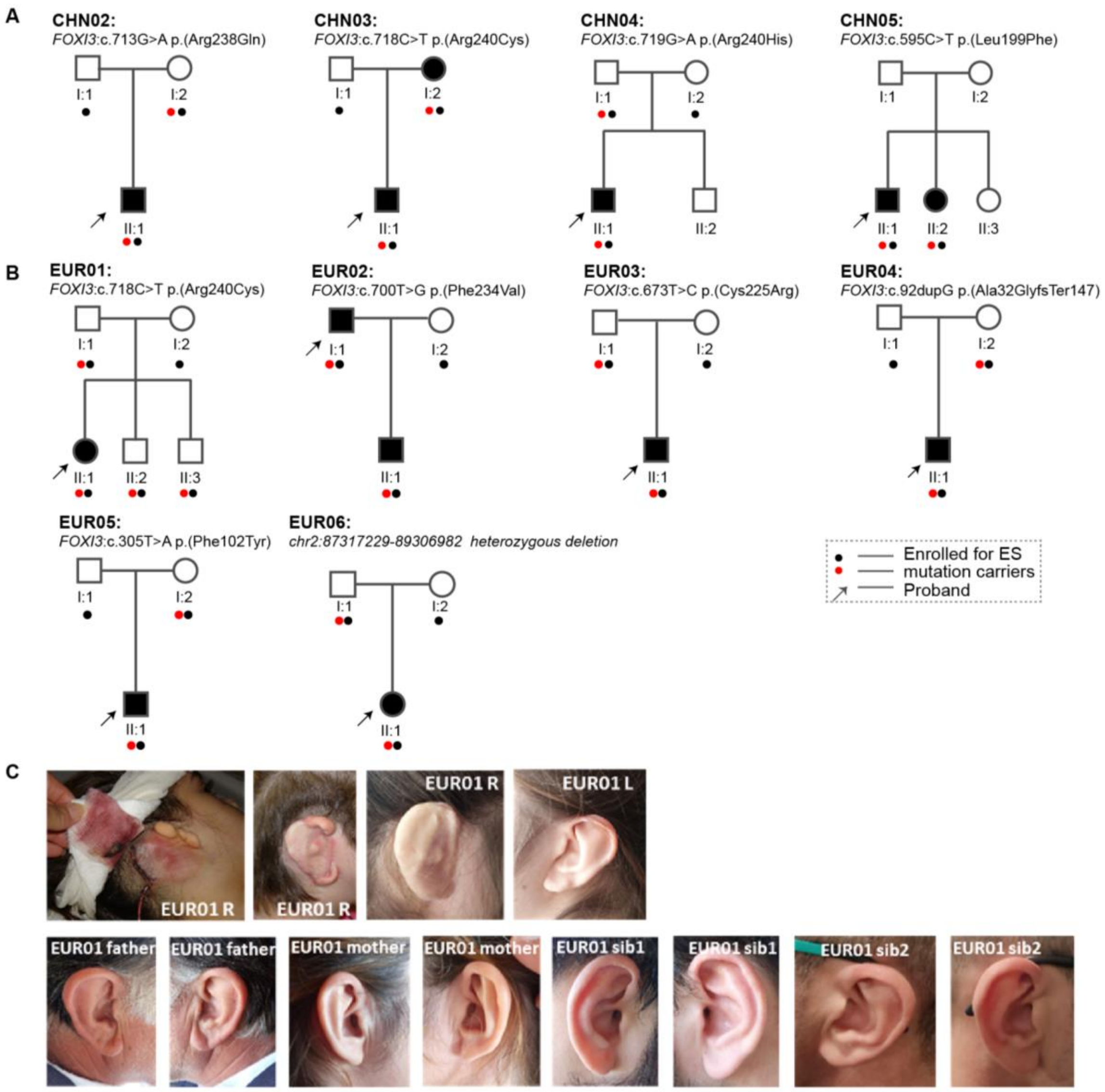

Supplementary Figure 2. Pedigrees of Chinese and European CFM families and ear phenotype of CFM patients. A: Pedigrees for the CHN02-05 families. B: Pedigrees for the European CFM families. The variants were detected by exome sequence (ES). C: The ear phenotype of family members of EUR01. The pictures of the right ear of patient EUR01 show the surgical reconstruction and the long-term outcome after the surgical procedure.

Supplementary Figure 3

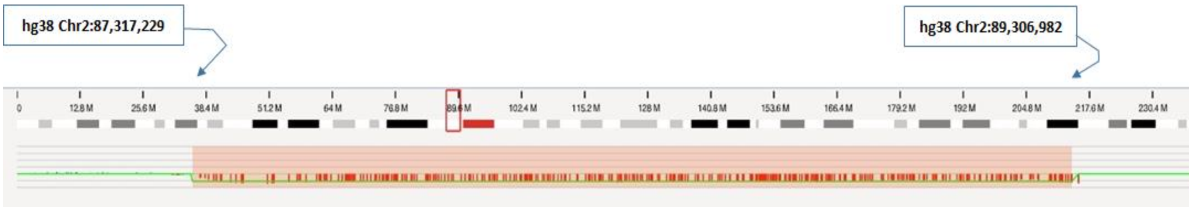

Supplementary Figure 3. Screenshot of the analysis of the Bionano experiment of sample EUR06 (SEA15421). The shaded area shows the extent of the heterozygous deletion that includes the *FOXI3* gene. The red box denotes the region of the deletion that contains *FOXI3*, and the red bar represents the centromeric region of chromosome 2.

Supplementary Figure 4

RS Number	Position (GRCh37)	Allele Frequencies	Haplotypes (South Asians)										Haplotypes (Europeans)								
rs11686866	chr2:88740296	G=0.718, A=0.282	G	A	G	A	G	A	G	G	G	G	G	G	A	G	G	G	A	G	A
rs12623469	chr2:88744777	C=0.718, A=0.282	C	A	C	A	C	C	C	A	C	C	C	C	A	C	A	C	A	C	A
rs67175137	chr2:88750971	G=0.722, C=0.278	G	C	G	C	G	G	G	C	G	G	G	G	C	G	G	G	C	G	G
rs11687776	chr2:88753364	A=0.721, G=0.279	A	G	A	G	A	A	A	G	A	A	A	A	G	A	A	A	G	A	A
rs56138227	chr2:88754736	T=0.722, C=0.278	T	C	T	C	T	T	T	C	T	T	T	T	C	T	T	T	C	T	T
rs66891658	chr2:88765532	G=0.723, A=0.277	G	A	G	A	G	G	G	A	G	G	G	G	A	G	G	G	A	G	G
rs56297047	chr2:88778804	C=0.639, T=0.361	C	T	T	C	C	C	C	T	C	C	C	T	T	C	C	C	C	C	C
rs61005807	chr2:88780398	G=0.639, T=0.361	G	T	T	G	G	G	G	T	G	G	G	T	T	G	G	G	G	G	G
rs6733320	chr2:88781819	G=0.526, A=0.474	G	A	A	G	A	G	A	A	A	A	G	A	A	G	G	A	G	A	G
rs371902356	chr2:88783938	G=0.529, -=0.471	G	-	-	G	-	G	-	-	-	-	G	-	-	G	G	-	G	-	G
rs6712707	chr2:88784093	A=0.636, C=0.364	A	C	C	A	A	A	A	C	A	A	A	C	C	A	A	A	A	A	A
rs11677224	chr2:88789815	T=0.583, C=0.417	T	C	C	T	T	T	C	C	C	C	T	C	C	T	T	C	T	T	T
rs62159788	chr2:88799514	C=0.583, A=0.417	C	A	A	C	C	C	A	A	A	A	C	A	A	C	C	A	C	C	C
rs11681923	chr2:88801960	C=0.617, G=0.383	C	G	G	C	C	C	G	G	C	C	C	G	G	C	C	C	C	C	C
rs10678096	chr2:88804446	-=0.583, TAAG=0.417	-	TAAG	TAAG	-	-	-	TAAG	TAAG	TAAG	TAAG	-	TAAG	TAAG	-	-	TAAG	-	-	-
rs56176904	chr2:88806758	A=0.617, G=0.383	A	G	G	A	A	A	G	G	A	A	A	G	G	A	A	A	A	A	A
rs34614579	chr2:88807984	-=0.583, AC=0.417	-	AC	AC	-	-	-	AC	AC	AC	AC	-	AC	AC	-	-	AC	-	-	-
rs6547780	chr2:88811787	T=0.578, C=0.422	T	C	C	T	T	T	C	C	C	C	T	C	C	T	T	C	T	T	T
rs7601037	chr2:88813394	T=0.578, C=0.422	T	C	C	T	T	T	C	C	C	C	T	C	C	T	T	C	T	T	T
rs4638798	chr2:88816123	T=0.612, C=0.388	T	C	C	T	T	T	C	C	T	T	T	C	C	T	T	T	T	T	T
rs7595875	chr2:88817092	T=0.612, C=0.388	T	C	C	T	T	T	C	C	T	T	T	C	C	T	T	T	T	T	T
rs10451671	chr2:88823509	T=0.583, C=0.417	T	C	C	T	T	T	C	C	C	C	T	C	C	T	T	C	T	T	T
rs56783496	chr2:88826219	G=0.616, A=0.384	G	A	A	G	G	G	A	A	G	G	G	A	A	G	G	G	G	G	G
rs7598916	chr2:88826598	C=0.601, A=0.399	C	A	A	C	C	C	A	A	A	C	C	A	A	C	C	C	C	C	C
rs7560217	chr2:88826943	T=0.616, C=0.384	T	C	C	T	T	T	C	C	T	T	T	C	C	T	T	T	T	T	T
rs62159807	chr2:88829371	G=0.616, A=0.384	G	A	A	G	G	G	A	A	G	G	G	A	A	G	G	G	G	G	G
rs6547781	chr2:88831691	C=0.616, T=0.384	C	T	T	C	C	C	T	T	C	C	C	T	T	C	C	C	C	C	C
rs56243080	chr2:88832146	T=0.583, C=0.417	T	C	C	T	T	T	C	C	C	C	T	C	C	T	T	C	T	T	T
rs111497808	chr2:88834130	T=0.583, C=0.417	T	C	C	T	T	T	C	C	C	C	T	C	C	C	T	C	T	T	T
		Haplotype Count	447	199	139	55	49	17	17	14	13	13	450	185	130	50	48	41	16	14	12
		Haplotype Frequency	0.4435	0.1974	0.1379	0.0546	0.0486	0.0169	0.0169	0.0139	0.0129	0.0129	0.4473	0.1839	0.1292	0.0497	0.0477	0.0408	0.0159	0.0139	0.0119

Supplementary Figure 4. Haplotypes of polymorphic sites around the *FOXI3* gene. The polymorphic sites are shown on the left. The various haplotypes are shown vertically. The haplotype shown by the red arrow in South Asians and Europeans respectively is mentioned in the text as the presumed trans haplotype that modifies the phenotypic severity of the *FOXI3*-related CFM. The frequency of the various haplotypes is shown at the bottom.

Supplementary Figure 5

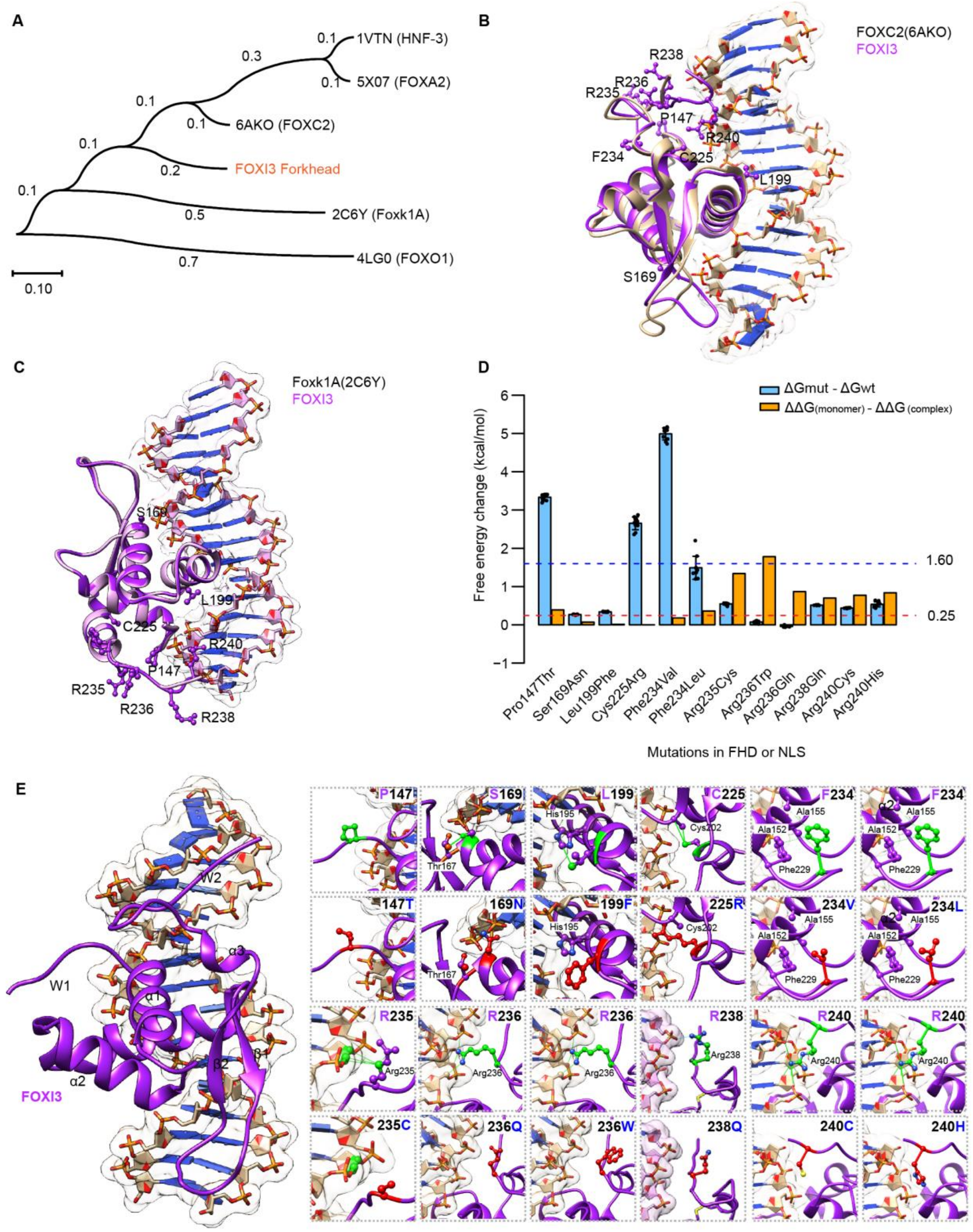

Supplementary Figure 5. Prediction on the effect of mutations on FOXI3 structure and stability. A: A phylogenetic tree of 6 members of the FOX family. The maximum likelihood tree is constructed according to the homology sequence of Forkhead Domain (FHD) and nuclear localization signal (NLS) by using MEGA. The branch length is labeled on each branch. The similarity between FOXI3

and other members is shown in brackets. B-C: Homology modeling for FOXI3 (in purple) is based on FOXC2 (B, 6AKO, in light golden) and Foxk1A (C, 2C6Y, in light pink). The positions of mutations are shown in the modeled crystal structure. The side chains of wild-type residues are represented. D: The prediction of mutations on protein stability and DNA binding ability. The prediction of free energy changes of each mutation on monomer and protein-DNA complex was replicated 10 times with default parameters. The blue bar indicates the average of the free energy changes (ΔGmut - ΔGwt) caused by each mutation (n = 10). Data are presented as the means ± s.d. The orange bar indicates the maximum difference of the free energy ($\Delta\Delta G_{(monomer)}$ -$\Delta\Delta G_{(complex)}$) between the prediction of each mutation based on the monomer and protein-DNA complex. Mutations with ΔGmut - ΔGwt > 1.6 kcal/mol were predicted as affecting the stability of FOXI3, while mutations with ΔGmut - ΔGwt < 1.6 kcal/mol and $\Delta\Delta G_{(monomer)}$ -$\Delta\Delta G_{(complex)}$ > 0.25 will affect the DNA binding ability[2,3]. Source data are provided as a Source Data file. E: The modeled 3D structure of FOXI3:DNA complex and the effects of mutations on the 3D structure. The 3D structure is modeled based on 6AKO (Left). The mutant protein caused by mutations from Pro147 to Arg236 is based on the predicted 3D structure of FOXI3 using 6AKO as a template, while Arg238 to Arg240 is based on the predicted 3D structure of FOXI3 using Foxk1A as a template. The wild-type residues are marked by green and mutant residues are marked by red. The interaction between residues is shown by the green line.

Supplementary Figure 6

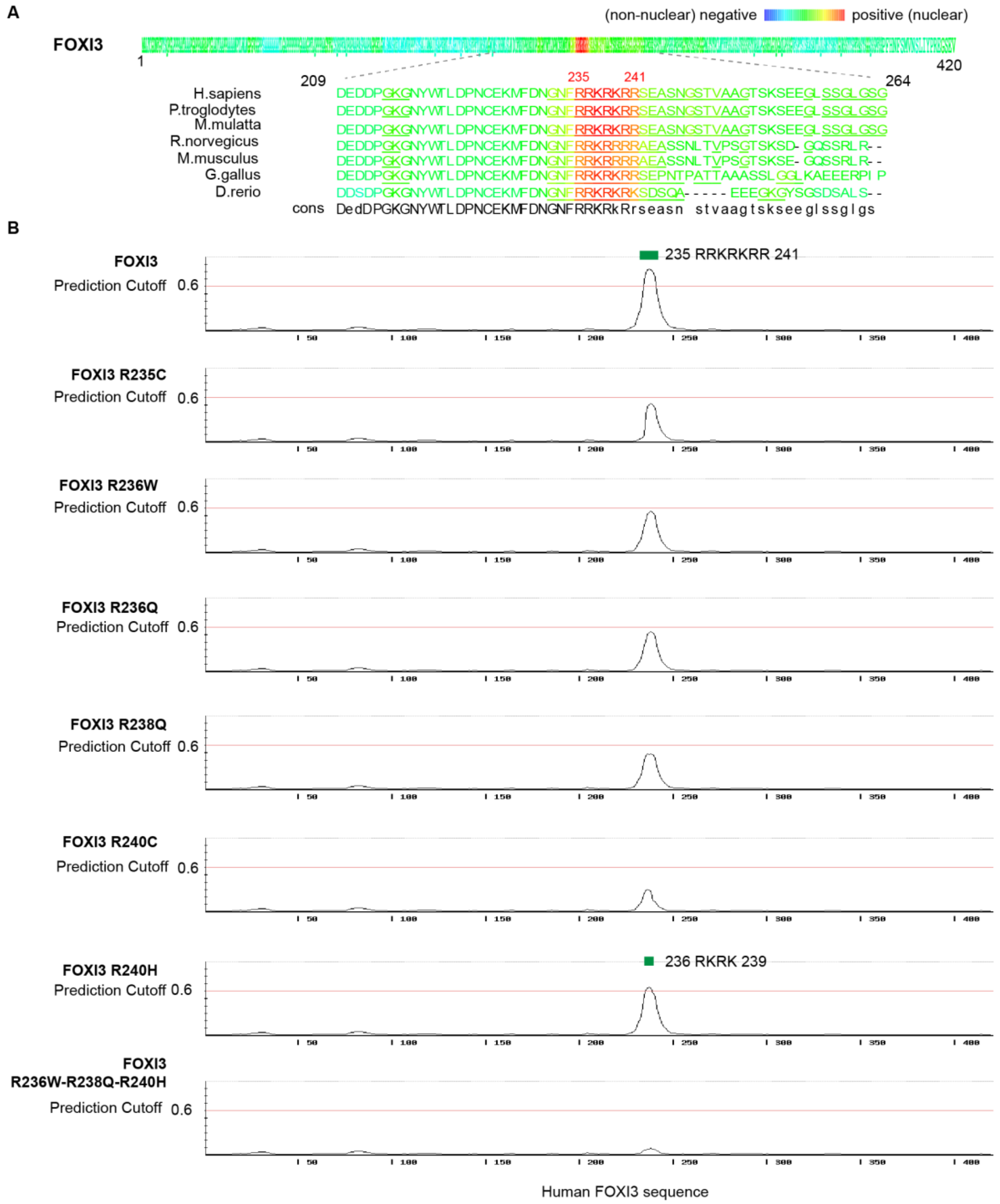

Supplementary Figure 6. The predicted structure of nuclear localization sequence (NLS) of FOXI3 and the effects of mutations in NLS. A: The prediction on the NLS of FOXI3 from 7 vertebrate species using Nucpred algorithm. The prediction on the whole human FOXI3 is shown at the top of the panel. The conservation analysis on the NLS across species is shown at the bottom of the panel. B: The prediction of the effect of mutations on NLS by NLStradamus algorithm[4]. The prediction cutoff value is using 0.6 as default and the green box shows the start and stop positions of predicted NLS.

Supplementary Figure 7

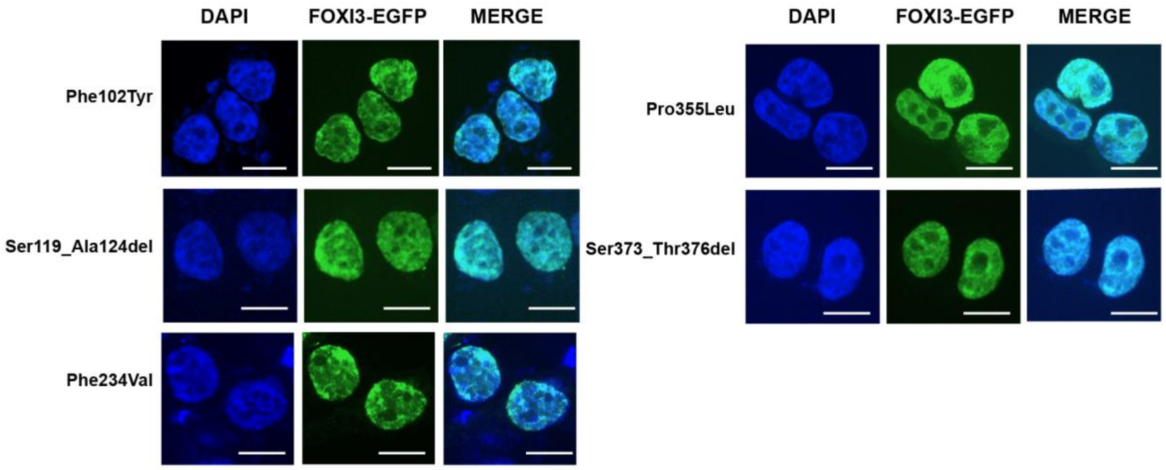

Supplementary Figure 7. The localization and distribution of FOXI3-EGFP fusion proteins with mutant IDR in HEK-293T cells. DAPI counterstain (blue) shows the location of the nucleus. EGFP (green fluorescent protein) shows the subcellular localization of the FOXI3-EGFP fusion protein. All mutations in IDRs seem not to affect the localization and distribution of FOXI3-EGFP. Scale bar = 10μm. Each experiment was performed in triplicate and repeated at least three times.

Supplementary Figure 8

A Forkhead box protein I3 [Homo sapiens] Length: 420
forkhead box protein I3 [Mus musculus] Length: 399

```
Human  1    MALYCGDNFGVYSQPGLPPPAATAAAPGAPPAARAPYGLADYAAPPAAAANPYLWLNGPG  60
            MALYCGDNF VYSQ          AAAPGAPP +RAPYGL+DYAAPPAAAANPYLWLNGPG
Mouse  1    MALYCGDNF-VYSQ--------PAAAPGAPPTSRAPYGLSDYAAPPAAAANPYLWLNGPG  51
Human  61   VGGPPSAAAAAAAAYLGAPPPPPPGAAAGPFLQPPPAAGTFGCSQRPFAQPAPAAPASP  120
            VGGP      A+AA+YLGAPPPPP  GAA GPFLQPP A GTF  +QR FAQP+ +APASP
Mouse  52   VGGP-----ASAASYLGAPPPPP--GAAPGPFLQPPAAPGTFAGAQRGFAQPSASAPASP  104
Human  121  AAPAGPGELGWLSMASREDLMKMVRPPYSYSALIAMAIQSAPERKLTLSHIYQFVADSFP  180
            A  A PGELGWLSMASREDLMKMVRPPYSYSALIAMAIQSAPERKLTLSHIYQFVAD+FP
Mouse  105  AGSAAPGELGWLSMASREDLMKMVRPPYSYSALIAMAIQSAPERKLTLSHIYQFVADNFP  164
Human  181  FYQRSKAGWQNSIRHNLSLNDCFKKVPRDEDDPGKGNYWTLDPNCEKMFDNGNFRRKRKR  240
            FYQRSKAGWQNSIRHNLSLNDCFKKVPRDEDDPGKGNYWTLDPNCEKMFDNGNFRRKR+R
Mouse  165  FYQRSKAGWQNSIRHNLSLNDCFKKVPRDEDDPGKGNYWTLDPNCEKMFDNGNFRRKRRR  224
Human  241  RSEASNGSTVAAGTSKSEEGLSSGLGSGVGGKPEEESPSTLLRPSHSPEPPEGTKSTASS  300
            R+EAS+   TV +GTSKS EG SS L    V GK E +SPS++LRPS SPEPPEGTKSTASS
Mouse  225  RAEASSNLTVPSGTSKS-EGQSSRL--RVSGKLEGDSPSSILRPSQSPEPPEGTKSTASS  281
Human  301  PGGPMLTSTPCLNTFFSSLSSLSV--SSSVSTQRALPGS-RHLGIQGAQLPSSGVFSPTS  357
            PG   LTSTPCLNTF S+ ++L+V  SSS+  QR LPGS RHLG  G QLPSS  F  TS
Mouse  282  PGASTLTSTPCLNTFLSTFNTLNVNSSSSMGNQRTLPGSRRHLG--GTQLPSS-TFPNTS  338
Human  358  ISEASADTLQLSNSTSNSTGQRSSYYSPFPASTSGGQSSPFSSPFHNFSMVNSLIYPREG  417
            + ++S D++QL   ST    + Q SSYY+PF   +SG QSSPFSSPF+NFSMVNSLIYPR+G
Mouse  339  VPDSSPDSMQL--STVGGSNQLSSYYNPFSGGSSGDQSSPFSSPFYNFSMVNSLIYPRDG  396
Human  418  SEV  420
            S++
Mouse  397  SDI  399
```

B

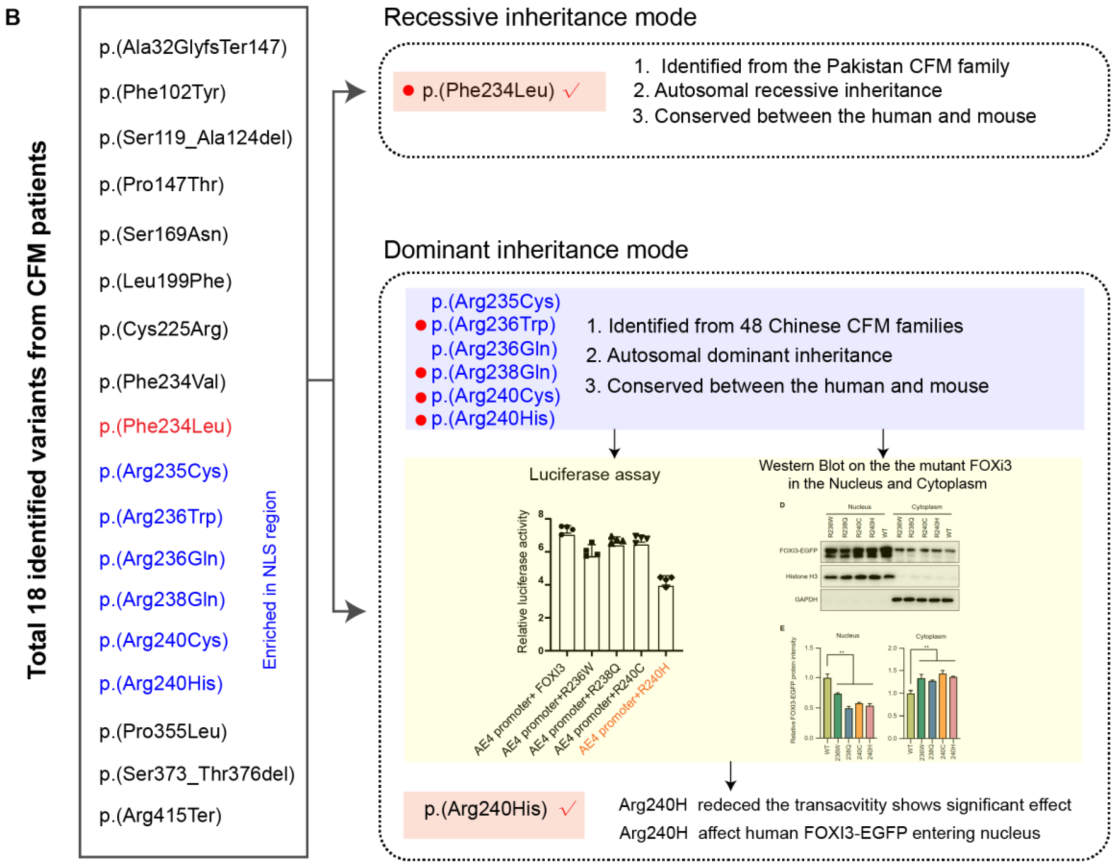

Supplementary Figure 8. Comparison of the protein sequences of human FOXI3 and mouse Foxi3. A: The orange and blue shaded sequences indicate the forkhead domain and NLS of human FOXI3 and mouse Foxi3, respectively. The homology similarity of FOXI3 protein sequence, forkhead domain, and NLS between human and mouse is 69.74%, 98.5%, and 85.7%, respectively. B: The

rationale for selecting p.(Phe234Leu) and p.(Arg240His) to produce mice models. The left panel shows all 18 variants identified in our cohorts. p.(Phe234Leu) is inherited in recessive mode and is marked by red letter. The variants within NLS are marked in blue. The right panel shows the rationale for selecting p.(Phe234Leu) and p.(Arg240His). Red dots indicate these variants are identified from pedigrees.

Supplementary Figure 9

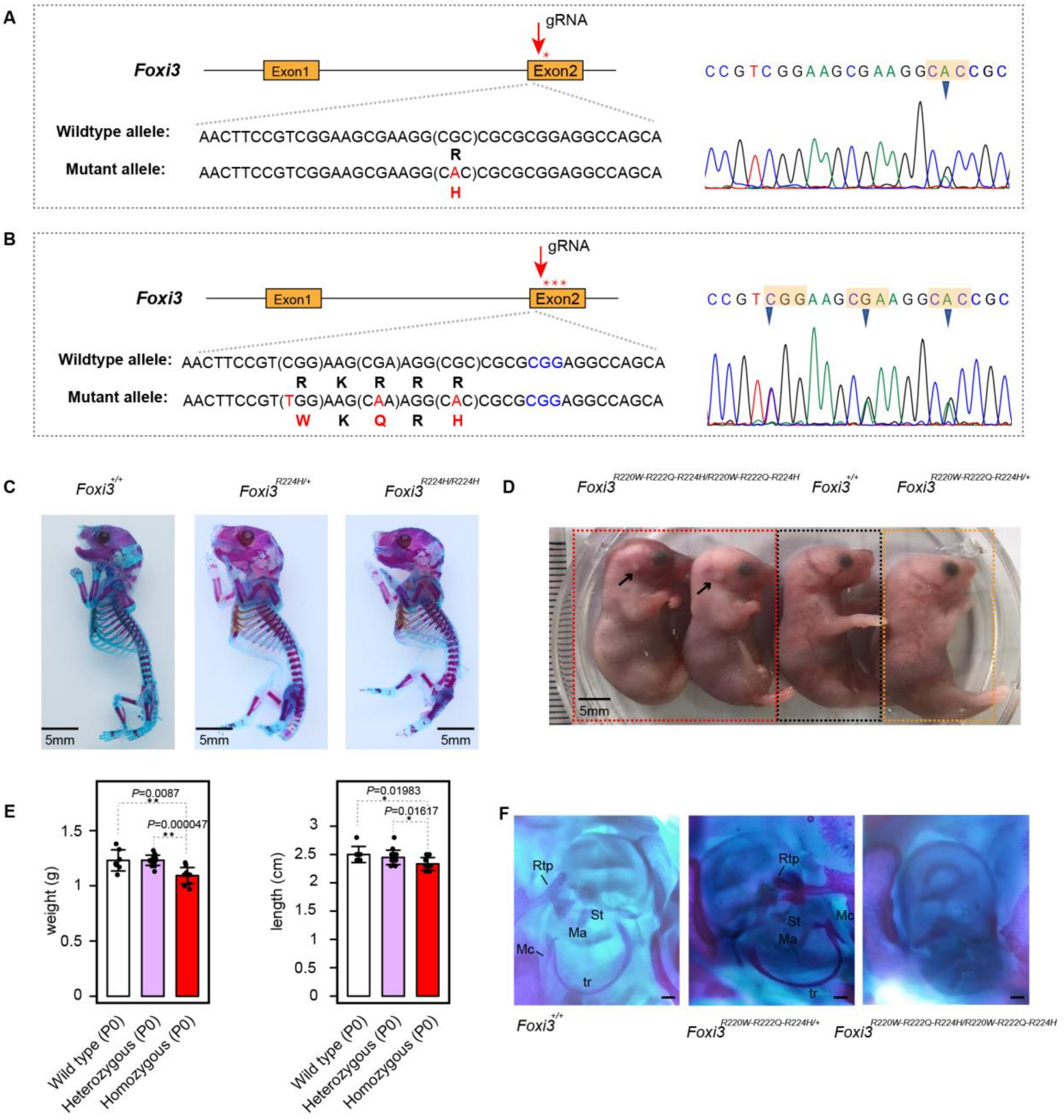

Supplementary Figure 9. The strategy of generating *Foxi3* mutant mice and the phenotypes of the mutant mice. A-B: Targeting strategy to generate *Foxi3*^{Arg224His} and *Foxi3*$^{R220W\text{-}R222Q\text{-}R224H}$ mutant mice. The mutant alleles of *Foxi3* are shown in red and the corresponding wild-type alleles are shown in black. Sanger sequencing is used to validate the genotype of the mutant mouse. C: Skeletal staining of wild-type, heterozygous, and homozygous *Foxi3*^{R224H} P0 mice (n = 3 for each genotype mice). D: The phenotypes of the homozygous and heterozygous *Foxi3*$^{R220W\text{-}R222Q\text{-}R224H}$ mice (P0)**.** The black arrow indicates the ear of the newborn homozygous mice. E: The statistics show the weight and length of the wild, heterozygous, and homozygous *Foxi3*$^{R220W\text{-}R222Q\text{-}R224H}$ newborn mice (P0; n = 7 for wild type, n = 15 for the heterozygous, and n = 11 for the homozygous). Significant differences between two groups (wild type and each mutant) were determined by unpaired Student's t-test (two-tailed). Data are presented as the means ± s.d. The asterisk (*) means the P-value < 0.05, (**) means the P-value < 0.01. Source data are provided as a Source Data file. F: The skeletal staining showed the differences

of the ear structure of the wild-type, heterozygous, and homozygous *Foxi3*$^{R220W\text{-}R222Q\text{-}R224H}$ mice at P0. P0: Postnatal day 0. Rtp: retrotympanic process. Ma: Malleus; St: Stapes; Mc: Meckel's cartilage; tr: tympanic ring. Scale bar = 100μm. Each experiment repeated at least three times.

Supplementary Figure 10

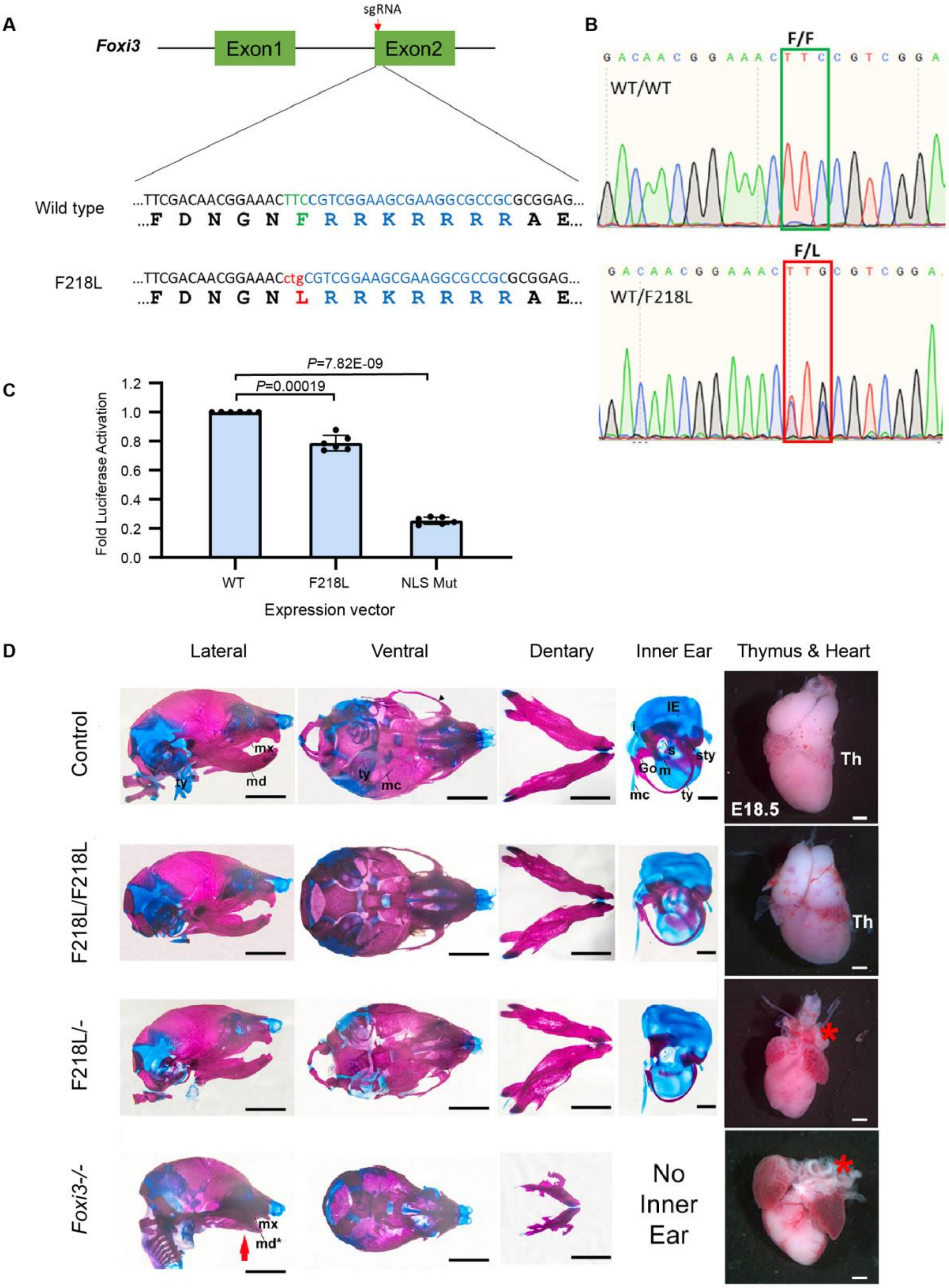

Supplementary Figure 10. **The *Foxi3*$^{Phe218Leu\ /\ Phe218Leu}$ mice.** A: Targeting strategy to generate *Foxi3*^{Phe218Leu} mutant mice. The sequence of interest and corresponding amino acid sequences are shown with the nuclear localization signal shown in blue. The *Foxi3*^{Phe218Leu} mutation is shown in red and the corresponding wild type sequence is shown in green. B: Validation of the CRISPR mutation by

Sanger sequencing. The top panel shows the sequence of wild type allele and the bottom panel shows the mutated allele generated by CRISPR targeting. The boxes denote the wild type and mutant sequences at position 218. C: The *Foxi3*^{Phe218Leu} variant has reduced transcriptional activity in a dual luciferase assay using the *AE4* promoter (*n* = 6/group). The p.(Phe218Leu) variant is compared to a mutant in which every amino acid was mutated to Ala for the nuclear localization signal (NLS), leading to a functionally null protein. We used luciferase reporter with *AE4* promoter along with blank FLAG as negative control and with wild-type *FOXI3* as positive control. Luciferase activity is shown as relative fold activation compared with control. Each experiment was performed in triplicate and was repeated at least two times. Significant differences between two groups (wild type and each mutant) were determined by unpaired Student's *t*-test (two-tailed). Data are presented as the means ± s.d.Source data are provided as a Source Data file. D: Phenotypic analysis of an allelic series of mouse *Foxi3* mutants. The *Foxi3* null mutant[5] is compared to a homozygous *Foxi3*^{Phe218Leu} / *Foxi3*^{Phe218Leu} mouse and a compound heterozygous F218L/null P0 mouse. Both *Foxi3*^{Phe218Leu} combinations have a normal jaw, inner ear and middle ear ossicles; however, F218L/null mice completely lack a thymus (asterisk). *Foxi3* null mice have a severely truncated jaw (red arrow), and completely lack the inner ear and thymus (asterisk). P0: Postnatal day 0. mx: Maxilla. md: Mandible. mc: Meckel's cartilage. m: Malleus. Go: Gonuim, ty: Tympanic ring. Scale bars = 2mm (lateral and ventral views), 1mm (dentary), 500µm (inner ear; thymus and heart). Each experiment repeated at least three times.

Supplementary Figure 11

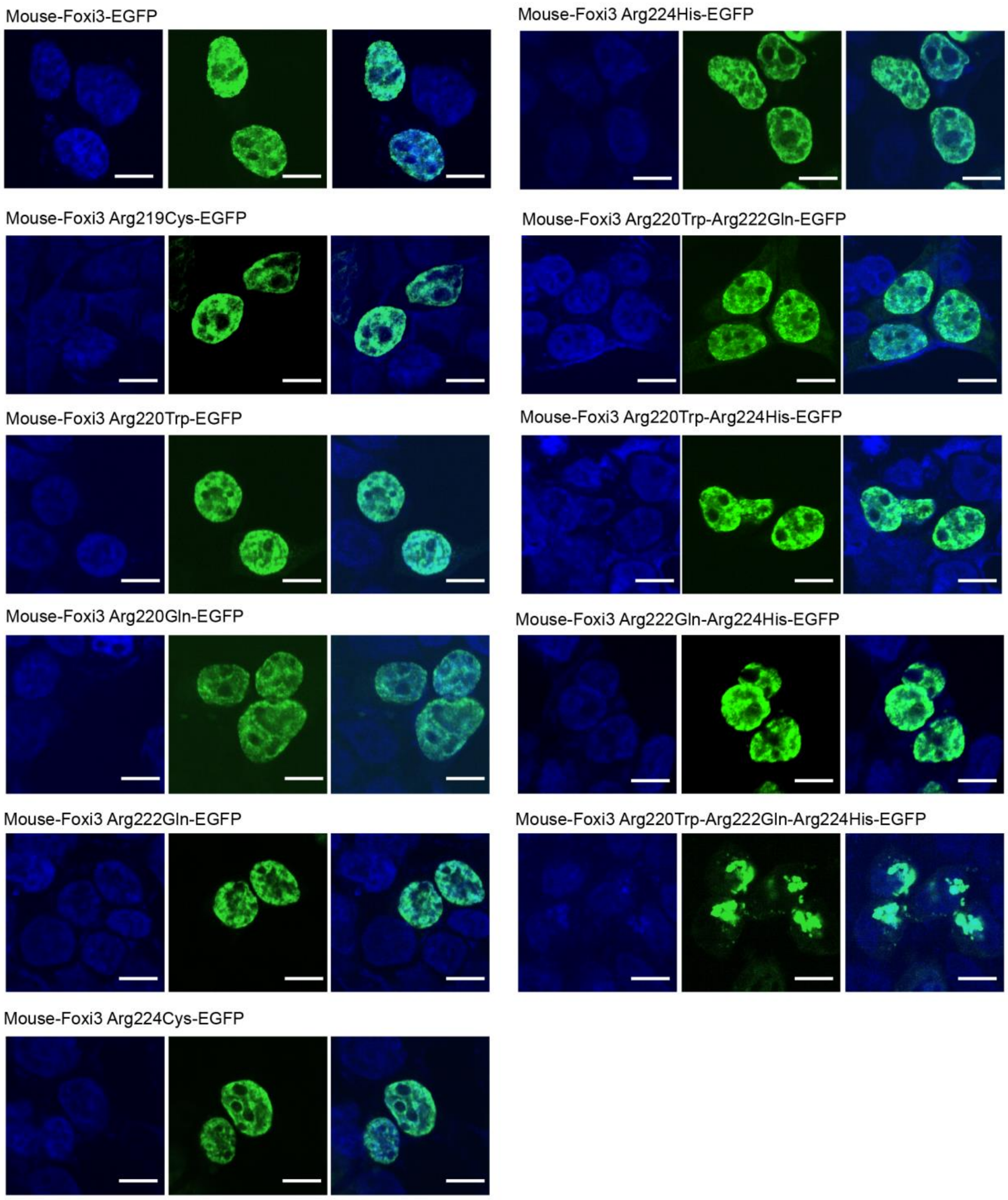

Supplementary Figure 11. The localization and distribution of wild-type or mutant mouse Foxi3-EGFP fusion proteins in HEK-293T cells. DAPI counterstain (blue) shows the location of the nucleus. EGFP (green fluorescent protein) shows the subcellular localization of the Foxi3-EGFP fusion proteins. Wild-type Foxi3 and single-point mutated Foxi3 are almost completely transferred into the nucleus and uniformly distributed. Mouse Foxi3 with triple mutation in NLS tends to block outside the nucleus. Scale bar = 10μm. Each experiment was performed in triplicate and repeated at least three times.

Supplementary Figure 12

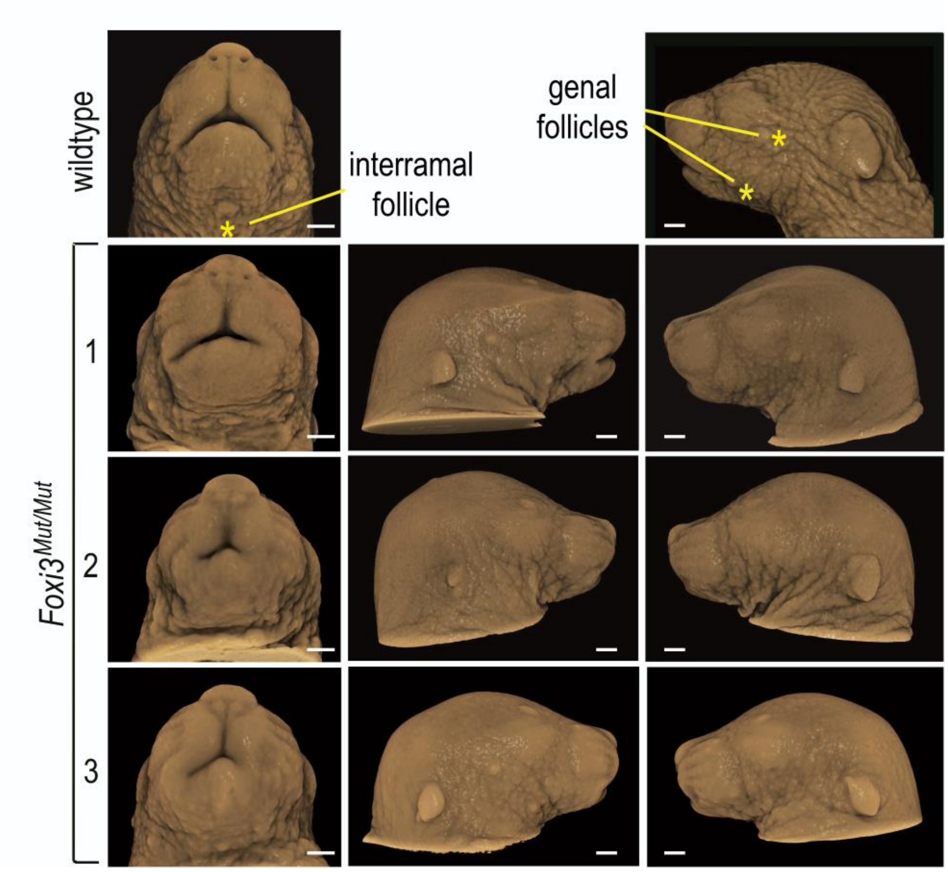

Supplementary Figure 12. P0 *Foxi3*$^{R220W\text{-}R222Q\text{-}R224H}$ homozygotes exhibit asymmetric and variable soft tissue facial phenotypes. Compared to wildtype littermates, *Foxi3*$^{R220W\text{-}R222Q\text{-}R224H}$ homozygotes (n = 3) all exhibit asymmetric microstomia and fully penetrant microtia but with asymmetry in severity. More severe presentations are associated with mandibular soft tissue transformations, including the presence of a line of mystacial whisker follicles on the mandible proper (see homozygote embryo in Fig 3E) and the absence of both the large lateral genal and central interramal whisker follicles (yellow asterisks on wild-type neonate). Note: The yellow line indicates bilateral absence of the genal follicles in *Foxi3*$^{Mut/Mut}$ embryo 2 and unilateral absence (left side) of the genal follicles in *Foxi3*$^{Mut/Mut}$ embryo 3. P0: Postnatal day 0. Scale bar = 1mm.

Supplementary References

1. Kuonen, D. Saddlepoint approximations for distributions of quadratic forms in normal variables. *Biometrika* 86, 929-935 (1999).
2. Alibés, A. *et al.* Using protein design algorithms to understand the molecular basis of disease caused by protein-DNA interactions: the Pax6 example. *Nucleic Acids Res* 38, 7422-31 (2010).
3. Williamson, K.A. *et al.* Recurrent heterozygous PAX6 missense variants cause severe bilateral microphthalmia via predictable effects on DNA-protein interaction. *Genet Med* 22, 598-609 (2020).
4. Nguyen Ba, A.N., Pogoutse, A., Provart, N. & Moses, A.M. NLStradamus: a simple Hidden Markov Model for nuclear localization signal prediction. *BMC Bioinformatics* 10, 202 (2009).
5. Edlund, R.K., Ohyama, T., Kantarci, H., Riley, B.B. & Groves, A.K. Foxi transcription factors promote pharyngeal arch development by regulating formation of FGF signaling centers. *Dev Biol* 390, 1-13 (2014).
