# Supplementary material for: FOXI3 pathogenic variants cause one form of craniofacial microsomia
Source: Nat Commun. 2023 Apr 11;14:2026. doi: 10.1038/s41467-023-37703-6 (PMC10090152; doi:10.1038/s41467-023-37703-6)
Supplement: Supplementary file 2 — Reporting Summary [file 41467_2023_37703_MOESM2_ESM.pdf]

nature portfolio

Corresponding author(s): Yong-Biao Zhang and Stylianos Antonarakis

Last updated by author(s): Nov 9, 2022

Reporting Summary

Nature Portfolio wishes to improve the reproducibility of the work that we publish. This form provides structure for consistency and transparency in reporting. For further information on Nature Portfolio policies, see our Editorial Policies and the Editorial Policy Checklist.

Statistics

For all statistical analyses, confirm that the following items are present in the figure legend, table legend, main text, or Methods section.

n/a	Confirmed	
☐	☒	The exact sample size (*n*) for each experimental group/condition, given as a discrete number and unit of measurement
☐	☒	A statement on whether measurements were taken from distinct samples or whether the same sample was measured repeatedly
☐	☒	The statistical test(s) used AND whether they are one- or two-sided<br>*Only common tests should be described solely by name; describe more complex techniques in the Methods section.*
☒	☐	A description of all covariates tested
☐	☒	A description of any assumptions or corrections, such as tests of normality and adjustment for multiple comparisons
☒	☐	A full description of the statistical parameters including central tendency (e.g. means) or other basic estimates (e.g. regression coefficient) AND variation (e.g. standard deviation) or associated estimates of uncertainty (e.g. confidence intervals)
☐	☒	For null hypothesis testing, the test statistic (e.g. *F*, *t*, *r*) with confidence intervals, effect sizes, degrees of freedom and *P* value noted<br>*Give P values as exact values whenever suitable.*
☒	☐	For Bayesian analysis, information on the choice of priors and Markov chain Monte Carlo settings
☒	☐	For hierarchical and complex designs, identification of the appropriate level for tests and full reporting of outcomes
☒	☐	Estimates of effect sizes (e.g. Cohen's *d*, Pearson's *r*), indicating how they were calculated

Our web collection on statistics for biologists contains articles on many of the points above.

Software and code

Policy information about availability of computer code

Data collection	No software was used
Data analysis	The commercial software used are mentioned in the methods

For manuscripts utilizing custom algorithms or software that are central to the research but not yet described in published literature, software must be made available to editors and reviewers. We strongly encourage code deposition in a community repository (e.g. GitHub). See the Nature Portfolio guidelines for submitting code & software for further information.

Data

Policy information about availability of data

All manuscripts must include a data availability statement. This statement should provide the following information, where applicable:

- Accession codes, unique identifiers, or web links for publicly available datasets
- A description of any restrictions on data availability
- For clinical datasets or third party data, please ensure that the statement adheres to our policy

The data of the exome sequences of individuals are not provided; however the authors could provide anonymously the .vcf files to interested scientists upon request.

Human research participants

Policy information about studies involving human research participants and Sex and Gender in Research.

Reporting on sex and gender	Sex and gender data have been collected.
Population characteristics	The populations of patients with Craniofacial Microsomia are of European and Chinese ancestry as described in the text and tables of the manuscript.
Recruitment	The cohorts of patients with CFM were recruited in genetics clinics on the basis of clinical findings compatible with the clinical diagnosis of CFM.
Ethics oversight	The project was approved by the Swiss Bioethics Committee of the University Hospitals and the Canton of Geneva (Protocol number: CER 11-036) and by local ethics committees and conducted under the Principles of Helsinki or an IRB approved protocol from USA. All the European samples were collected after informed consent from the patients or the parents. Consent for publication of photographs was given after a separate explanation and request. The Chinese subjects were collected from the Beijing Tongren Hospital, Capital Medical University and Plastic Surgery Hospital of the Peking Union Medical College, China. Exome sequencing and target-capture sequencing were performed on 159 enrolled individuals from 48 CFM families and 498 sporadic CFM patients, respectively. All participants or their guardians signed informed consent forms for biological investigation. The project was reviewed and approved by the Ethics Committees of the School of Biological Science and Medical Engineering, Beihang University, the Tongren Hospital, Capital Medical University, and the Plastic Surgery Hospital of the Peking Union Medical College and conducted under the Principles of Helsinki.

Note that full information on the approval of the study protocol must also be provided in the manuscript.

Field-specific reporting

Please select the one below that is the best fit for your research. If you are not sure, read the appropriate sections before making your selection.

☒ Life sciences ☐ Behavioural & social sciences ☐ Ecological, evolutionary & environmental sciences

For a reference copy of the document with all sections, see nature.com/documents/nr-reporting-summary-flat.pdf

Life sciences study design

All studies must disclose on these points even when the disclosure is negative.

Sample size	Craniofacial Microsomia (CFM) is a rare genetic developmental syndrome. we have collected a sample of 670 affected individuals and members of their families. This sample size is the largest reported in the biomedical literature.
Data exclusions	No data were excluded
Replication	All relevant data from the exome and genome sequencing were validated by Sanger sequencing of PCR products.
Randomization	There are no experimental groups other than affected and non affected individuals by clinical evaluations.
Blinding	There was not blinding in this rather diagnostic study.

Reporting for specific materials, systems and methods

We require information from authors about some types of materials, experimental systems and methods used in many studies. Here, indicate whether each material, system or method listed is relevant to your study. If you are not sure if a list item applies to your research, read the appropriate section before selecting a response.

Materials & experimental systems

n/a	Involved in the study	
☐	☒	Antibodies
☐	☒	Eukaryotic cell lines
☒	☐	Palaeontology and archaeology
☐	☒	Animals and other organisms
☐	☒	Clinical data
☒	☐	Dual use research of concern

Methods

n/a	Involved in the study	
☒	☐	ChIP-seq
☒	☐	Flow cytometry
☒	☐	MRI-based neuroimaging

Antibodies

Antibodies used	The primary antibodies included anti-EGFP (Abcam, Cambridge, UK), nuclear protein internal control anti-H3 (Abcam, Cambridge, UK), cytoplasmic protein internal control anti-GAPDH (Abcam, Cambridge, UK).
Validation	Validation was done by the manufacturer Abcam, Cambridge, UK.

Eukaryotic cell lines

Policy information about cell lines and Sex and Gender in Research

Cell line source(s)	HEK-293T
Authentication	The HEK-293T was authenticated by the Nest Scientific, Wuxi, Jiangsu, China
Mycoplasma contamination	Cell lines were tested negative for Mycoplasma
Commonly misidentified lines (See ICLAC register)	There are no misidentified lines

Animals and other research organisms

Policy information about studies involving animals; ARRIVE guidelines recommended for reporting animal research, and Sex and Gender in Research

Laboratory animals	Mice C57BL/6
Wild animals	N/A
Reporting on sex	There are no sex differences in the resulting phenotypes
Field-collected samples	N/A
Ethics oversight	All animal experiments were performed under the approval of the Animal Care Committee in Beihang University and the Institutional Animal Care and Use Committee at Baylor College of Medicine.

Note that full information on the approval of the study protocol must also be provided in the manuscript.

Clinical data

Policy information about clinical studies

All manuscripts should comply with the ICMJE guidelines for publication of clinical research and a completed CONSORT checklist must be included with all submissions.

Clinical trial registration	N/A
Study protocol	The subjects were recruited as part of diagnostic studies
Data collection	The recruitment of patients with CFM was done over a period of 4 years
Outcomes	N/A
